# Preamplification techniques for real-time RT-PCR analyses of endomyocardial biopsies

**DOI:** 10.1186/1471-2199-9-3

**Published:** 2008-01-14

**Authors:** Michel Noutsias, Maria Rohde, Andrea Block, Katrin Klippert, Olga Lettau, Katja Blunert, Michael Hummel, Uwe Kühl, Hans Lehmkuhl, Roland Hetzer, Ursula Rauch, Wolfgang Poller, Matthias Pauschinger, Heinz P Schultheiss, Hans D Volk, Katja Kotsch

**Affiliations:** 1 Department of Cardiology and Pneumonology, Charité – Universitätsmedizin Berlin, Campus Benjamin Franklin, 12200 Berlin, Germany; 2 Institute of Medical Immunology, Charité – Universitätsmedizin Berlin, Campus Mitte, 10117 Berlin, Germany; 3 Department of Pathology, Charité – Universitätsmedizin Berlin, Campus Benjamin Franklin, 12200 Berlin, Germany; 4 Deutsches Herzzentrum Berlin, 13353 Berlin, Germany

## Abstract

**Background:**

Due to the limited RNA amounts from endomyocardial biopsies (EMBs) and low expression levels of certain genes, gene expression analyses by conventional real-time RT-PCR are restrained in EMBs. We applied two preamplification techniques, the TaqMan^® ^PreAmp Master Mix (T-PreAmp) and a multiplex preamplification following a sequence specific reverse transcription (SSRT-PreAmp).

**Results:**

T-PreAmp encompassing 92 gene assays with 14 cycles resulted in a mean improvement of 7.24 ± 0.33 Ct values. The coefficients for inter- (1.89 ± 0.48%) and intra-assay variation (0.85 ± 0.45%) were low for all gene assays tested (<4%). The PreAmp uniformity values related to the reference gene CDKN1B for 91 of the investigated gene assays (except for CD56) were -0.38 ± 0.33, without significant differences between self-designed and ABI inventoried Taqman^® ^gene assays. Only two of the tested Taqman^® ^ABI inventoried gene assays (HPRT-ABI and CD56) did not maintain PreAmp uniformity levels between -1.5 and +1.5. In comparison, the SSRT-PreAmp tested on 8 self-designed gene assays yielded higher Ct improvement (9.76 ± 2.45), however was not as robust regarding the maintenance of PreAmp uniformity related to HPRT-CCM (-3.29 ± 2.40; p < 0.0001), and demonstrated comparable intra-assay CVs (1.47 ± 0.74), albeit higher inter-assay CVs (5.38 ± 2.06; p = 0.01). Comparing EMBs from each 10 patients with dilated cardiomyopathy (DCM) and inflammatory cardiomyopathy (DCMi), T-PreAmp real-time RT-PCR analyses revealed differential regulation regarding 27 (30%) of the investigated 90 genes related to both HPRT-CCM and CDKN1B. Ct values of HPRT and CDKN1B did not differ in equal RNA amounts from explanted DCM and donor hearts.

**Conclusion:**

In comparison to the SSRT-PreAmp, T-PreAmp enables a relatively simple workflow, and results in a robust PreAmp of multiple target genes (at least 92 gene assays as tested here) by a mean Ct improvement around 7 cycles, and in a lower inter-assay variance in RNA derived from EMBs. Preliminary analyses comparing EMBs from DCM and DCMi patients, revealing differential regulation regarding 30% of the investigated genes, confirm that T-PreAmp is a suitable tool to perform gene expression analyses in EMBs, expanding gene expression investigations with the limited RNA/cDNA amounts derived from EMBs. CDKN1B, in addition to its function as a reference gene for the calculation of PreAmp uniformity, might serve as a suitable housekeeping gene for real-time RT-PCR analyses of myocardial tissues.

## Background

Dilated cardiomyopathy (DCM) is a heterogeneous disease of multifactorial etiopathogenesis. However, investigations on endomyocardial biopsies (EMBs) have revealed that chronic T-cell infiltration (inflammatory cardiomyopathy/DCMi), induced and maintained by cardiotropic viral infections, as well as by secondary autoimmune pathways, is identifiable in a significant proportion of patients presenting with DCM [1-6]. In addition, functional characterization of infiltrates and expression investigations of further immunocompetent factors may be decisively important for a profound understanding of the pathogenesis of DCMi, as well as for immunomodulatory treatment strategies [5,7-13]. Furthermore, expression of genes involved in general heart failure pathways such as the remodeling of the extracellular matrix is altered in EMBs from DCM patients [14]. Gene expression investigations of EMBs are promising approaches for the investigation novel pathogenic pathways involved in DCM/DCMi [15], and may reveal profiles with prognostic impact for the natural course of the disease as well as under immunomodulatory treatment [4,11-13,16].

Real-time RT-PCR is a sensitive, precise and reproducible tool to determine gene expression in tissues. However, the RNA amounts extractable from EMBs are low, thereby limiting the number of analyzable target genes by real-time RT-PCR. Preamplification (PreAmp) techniques may enhance sensitivity of real-time RT-PCR especially for low abundance genes, and can establish substantially higher cDNA amounts, which can then expand the number of the analyzable target genes. However, maintenance of gene expression profiles as well as a broad applicability to many target genes with a feasible additional effort is a further intriguing aspect of PreAmp procedures.

We here report the application of the TaqMan^® ^PreAmp Master Mix technique (T-PreAmp) to EMBs' cDNA, which, as in PBMCs' cDNA, enables a robust PreAmp of multiple pooled TaqMan^® ^ABI inventoried gene expression assays and primers of self-designed gene expression assays, including wobbled primer designs, with acceptable PreAmp uniformity values (except for 2 of the investigated gene assays), and low intra- and inter-assay variance coefficients. We compared this technique with a sequence-specific reverse transcription (SSRT) technique followed by a multiplex PreAmp step (SSRT-PreAmp) in accordance to the strategy by Peixoto et al. [17] in a set of self-designed 7 gene assays for T-cell specific genes, which performed with a partly higher PreAmp efficacy (improvement of Ct values), however resulted in substantial variations of PreAmp uniformity, and higher inter-assay variation coefficients (CVs), and moreover did not offer the possibility of expanding the possible real-time RT-PCR analyses out of the limited EMBs' cDNA. Furthermore, we show preliminary data on the differential expression of 27 out of 90 (30%) investigated target genes in patients with EMBs-confirmed DCMi compared with EMBs from DCM patients. Moreover, we provide evidence that CDKN1B (cyclin-dependent kinase inhibitor 1B) as well as HPRT (hypoxanthine-guanine phosphoribosyltransferase) is not differentially expressed in DCM versus donor hearts, and may therefore be a relevant housekeeping gene candidate for real-time RT-PCR analyses of myocardial tissues.

## Results

### PreAmp real-time RT-PCR from PBMCs' cDNA

#### T-PreAmp

Albeit the Ct values obtained from cDNA from both PBMCs and EMBs in conventional direct real-time PCR were virtually equivalent (difference around 0.3 Ct values) with respect to the hypoxanthine-guanine phosphoribosyltransferase (HPRT) gene assay designed by the Institute of Medical Immunology at the Charité-Campus Mitte (HPRT-CCM) and the ABI inventoried Taqman^® ^gene assay for HPRT (HPRT-ABI) in initial investigations, T-PreAmp cDNA revealed differences in PreAmp uniformity values of these two HPRT gene assays. Whereas the Ct improvement for HPRT-CCM was 7.2, the gene assay HPRT-ABI achieved a Ct improvement of only 4.9 cycles. This resulted in a PreAmp uniformity related to CDKN1B of 0.82 for HPRT-CCM and 3.14 for HPRT-ABI, respectively. In light of this minor T-PreAmp performance of the HPRT-ABI gene assay, we used further on only the HPRT-CCM as housekeeping gene assay. Direct real-time PCR from the pooled PBMCs' cDNA revealed a mean Ct value 31.28 ± 3.47 (range: 24.75 to 38.93), and in T-PreAmp cDNA a mean Ct value of 23.91 ± 3.46 (range: 16.92 to 31.85) for the investigated gene assays excluding CD56. T-PreAmp yielded a mean improvement of 7.37 ± 0.51 cycles (range: 6.35 to 8.90) for the investigated gene assays (p < 0.0001 compared with the Ct values derived from the direct real-time RT-PCR). There was no traceable gene expression neither in the non-preamplified nor in the T-PreAmp cDNA with respect to IL-5, IL-10, IL-17, IL-27, IFN-b, CYR61, CXCL14, CX3CL1, APN, GRAIL, collagen types I, III and IV, laminin, MMP3, TIMP-4, GDF15 and CD62E. Considering the threshold Ct for low expression levels published by Canales et al. [18], 13/78 (17%) of the target genes showed Ct values ± 35 in direct real-time RT-PCR. The mean PreAmp uniformity for the investigated gene assays in PBMCs (excluding CD56) related to CDKN1B was 0.24 ± 0.50, ranging from -1.26 to 1.29, and the PreAmp uniformity in PBMCs related to HPRT-CCM was 0.39 ± 0.54, ranging from -1.06 to 1.49, respectively. There was no significant difference between the self-designed (mean Ct improvement: 7.46 ± 0.49 cycles; PreAmp uniformity related to CDKN1B: 0.23 ± 0.47, and related to HPRT-CCM: 0.34 ± 0.56, respectively) and the Taqman^® ^ABI inventoried gene assays (mean Ct improvement: 7.38 ± 0.53 cycles; PreAmp uniformity related to CDKN1B: 0.25 ± 0.53, and related to HPRT-CCM: 0.45 ± 0.52, respectively; p each >0.05; excluding CD56). With respect to CD56, PreAmp uniformity related to CDKN1B was 3.63, and 2.82 related to HPRT-CCM, respectively. There was no association between the PreAmp uniformity related to CDKN1B of target gene Ct values in direct real-time RT-PCR < 35 (0.27 ± 0.43) or ≥ 35 (0.05 ± 0.78). The same was true for the PreAmp uniformity values related to HPRT-CCM (Ct < 35: 0.45 ± 0.46; Ct ≥ 35: 0.17 ± 0.86).

**Figure 1:**
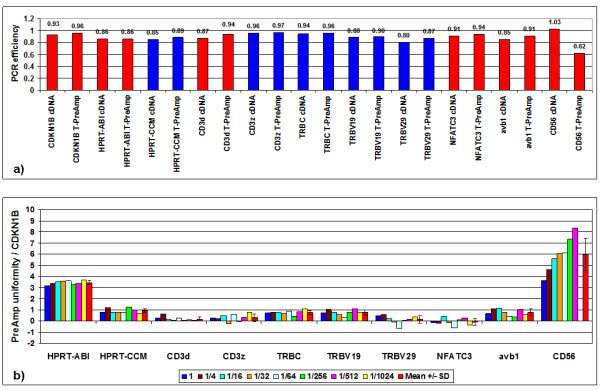
**PCR efficiency and PreAmp uniformity in PBMCs using T-PreAmp**. a) Comparison of PCR efficiency of the investigated candidate gene assays calculated from PBMCs' cDNA (25 ng/μl) and 7 dilution steps (up to 1:1,024) by direct (cDNA) and T-PreAmp real-time RT-PCR (T-PreAmp). Taqman^® ^ABI inventoried gene assays are indicated by red bars, self-designed gene assays designed by coworkers of the Charité are indicated by blue bars. b) PreAmp uniformity of the investigated candidate gene assays at 7 dilution steps from 25 ng/μl PBMCs' cDNA (1) to 1:1,024. The red bars indicate the means ± SD of the 8 reactions for each gene assay.

In light of the disproportionate PreAmp uniformities of HPRT-CCM and HPRT-ABI, whilst yielding comparable Ct values in conventional real-time RT-PCR, we hypothesized that PCR efficiency during PreAmp might be a pivotal issue. The comparison of the two gene assays showing minor PreAmp performance (HPRT-ABI and CD56) to candidate (self-designed and Taqman^® ^ABI inventoried) gene assays with T-PreAmp uniformities ranging between -1.5 and +1.5 (HPRT-CCM, CD3d, CD3z, TRBC, TRBV19, TRBV29, NFATC3 and avb1) over serial dilutions of PBMCs' cDNA revealed a non-significant tendency for increased PCR efficiency by T-PreAmp (0.92 ± 0.04 versus 0.89 ± 0.05 by direct real-time RT-PCR; excluding CD56; Figure 1a). This was also true for HPRT-ABI. PreAmp uniformity for these gene assays except for CD56 showed comparable PreAmp uniformities over the serial cDNA dilutions (Figure 1b). The CD56 gene assay, however, demonstrated a substantially decreased PCR efficiency by T-PreAmp compared with direct real-time RT-PCR (0.62 versus 1.03; Figure 1a), and a gradually increasing PreAmp uniformity at serial cDNA dilutions to 8.37 at the ratio 1:512 (no expression at 1:1,024; Figure 1b).

### SSRT-PreAmp (sequence-specific reverse transcription followed by a multiplex PreAmp)

Using the SSRT-PreAmp system on HPRT-CCM as housekeeping gene and further 7 T-cell related self-designed gene assays, a mean improvement of 9.26 ± 1.94 cycles (range: 7.09–11.9) was obtained. The measured Ct values in direct real-time RT-PCR were 29.99 ± 3.84 (range: 24.18–37.36), and in SSRT-PreAmp 20.73 ± 3.81 (range: 16.40–28.53; p < 0.0001 compared with the Ct values derived from the direct real-time RT-PCR). Considering the threshold Ct for low expression levels published by Canales et al. [18], 1/7 (14%) of the target genes showed Ct values ≥ 35 in direct real-time RT-PCR. The mean PreAmp uniformity for the investigated gene assays related to HPRT-CCM was -2.49 ± 2.04 (range: -4.81–0), which was significantly (p < 0.0001) lower compared with the T-PreAmp data. The following gene assays were below a PreAmp uniformity of -1.5: CD3z, IFNg, T-bet and Perforin. There was no association between the PreAmp uniformity related to HPRT-CCM of target gene Ct values in direct real-time RT-PCR < 35 (-2.61 ± 0.9) or ≥ 35 (-1.75 ± 2.2).

### PreAmp real-time RT-PCR from EMBs

#### T-PreAmp

T-PreAmp of EMBs' cDNA with the investigated target gene assays resulted in a mean Ct improvement of 6.86 ± 0.13 (range: 6.65 – 7.07) for CDKN1B, and in mean improvement of 7.16 ± 0.14 Ct values (range: 6.91 – 7.39; p < 0.0001 compared with the Ct values derived from the direct real-time RT-PCR) for HPRT-CCM, respectively. The mean PreAmp uniformity for HPRT-CCM related to CDKN1B was -0.30 ± 0.05 (range: -0.37 to -0.21). The mean ΔΔCt [PreAmp cDNA] – ΔCt [cDNA] for HPRT-CCM and CDKN1B was 0.30 ± 0.05 (range: 0.22 to 0.37). There was a high association between the Ct values of HPRT-CCM and CDKN1B both in cDNA (Spearman ρ: 0.86, p < 0.0001) and in PreAmp cDNA (Spearman ρ: 0.83, p < 0.0001). Moreover, the ΔCt values of CDKN1B and HPRT-CCM of [cDNA] versus [PreAmp cDNA] showed a high correlation (Spearman ρ: 0.99, p < 0.0001). The inter-assay CVs were 1.85% for CDKN1B, and 1.93% for HPRT-CCM, respectively.

**Figure 2:**
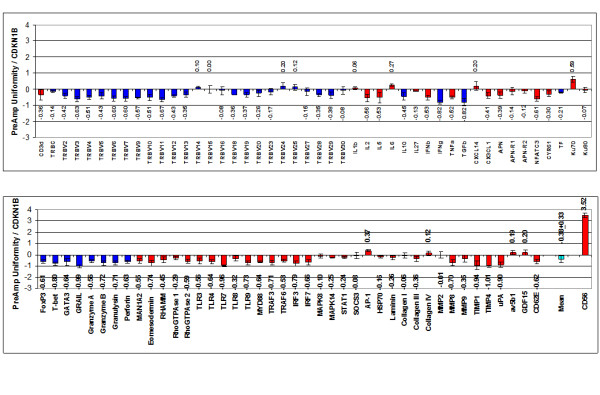
**PreAmp uniformity in EMBs using T-PreAmp related to CDKN1B**. Taqman^® ^ABI inventoried gene assays are indicated by red bars, self-designed gene assays designed by coworkers of the Charité are indicated by blue bars. Values are displayed as means ± SD. The turquoise (second to last) bar indicates the PreAmp uniformity mean ± SD from all gene assays excluding CD56, which is indicated by the last bar.

**Figure 3:**
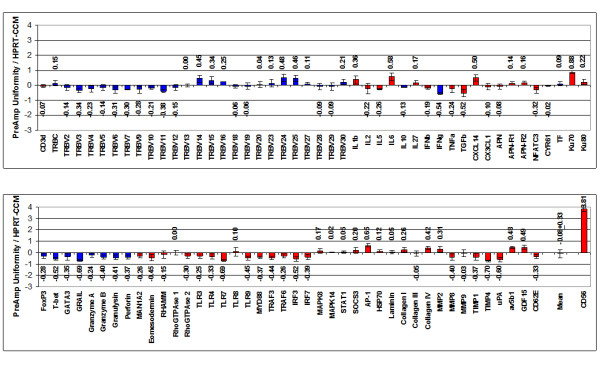
**PreAmp uniformity in EMBs using T-PreAmp related to HPRT-CCM**. Taqman^® ^ABI inventoried gene assays are indicated by red bars, self-designed gene assays designed by coworkers of the Charité are indicated by blue bars. Values are displayed as means ± SD. The turquoise (second to last) bar indicates the PreAmp uniformity mean ± SD from all gene assays excluding CD56, which is indicated by the last bar.

**Figure 4:**
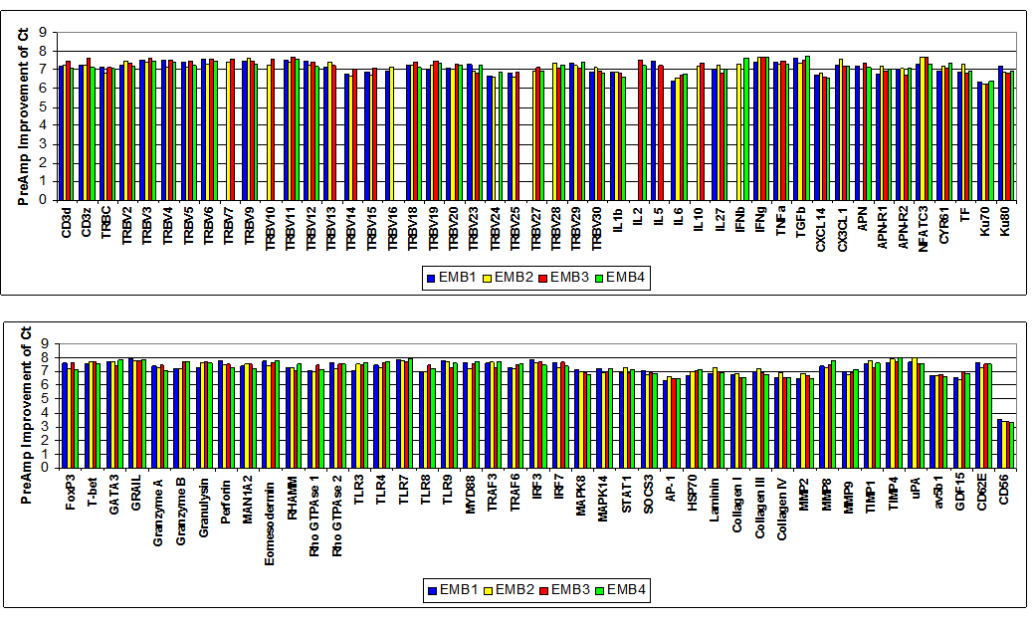
**Inter-assay variance of Ct improvement in EMBs using T-PreAmp**. Ct improvement achieved by T-PreAmp from 4 different EMBs (illustrated in 4 different colors) for each gene assay. The individual Ct values of CDKN1B and HPRT-CCM are not shown, since they were determined in all 38 EMBs.

**Figure 5:**
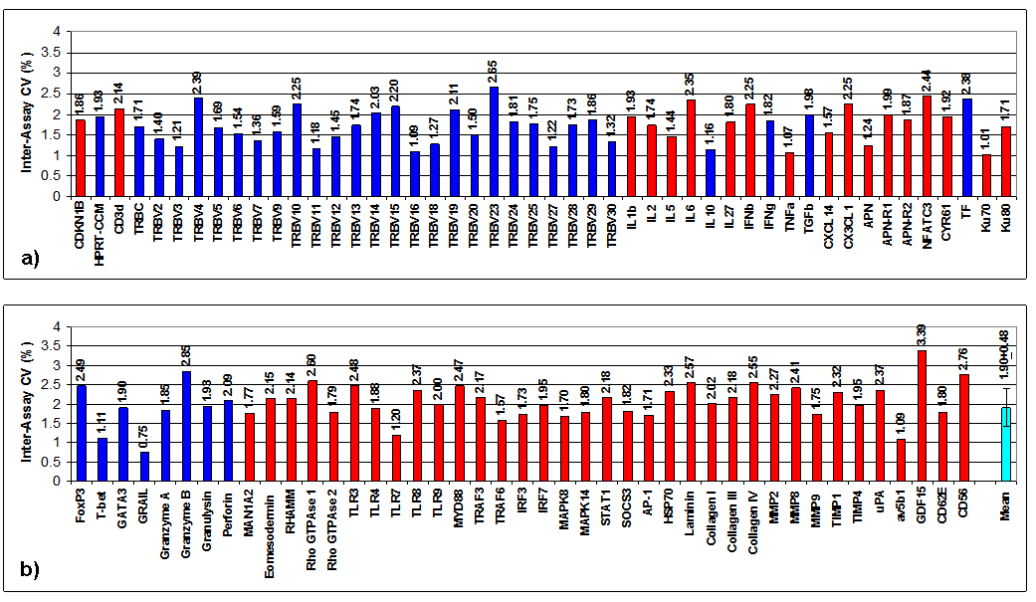
**Inter-assay CV of Ct improvement in EMBs using T-PreAmp**. Inter-assay CV of Ct improvement by T-PreAmp derived from 4 different EMBs. Taqman^® ^ABI inventoried gene assays are indicated by red bars, self-designed gene assays designed by coworkers of the Charité are indicated by blue bars. The turquoise bar indicates the mean ± SD of the investigated gene assays.

With respect to the investigated target gene assays (except for CD56), T-PreAmp yielded a mean improvement of 7.24 ± 0.33 Ct values (range: 6.31 to 7.84). The CD56 gene assay demonstrated a mean Ct improvement of 3.37 ± 0.09. The PreAmp uniformity of the investigated gene assays (except for CD56) related to CDKN1B was -0.38 ± 0.33 (range: -1.01 to 0.59; Figure 2), and related to HPRT-CCM -0.08 ± 0.33, (range: -0.70 to 0.88; Figure 3), respectively. As in cDNA from PBMCs, there was no significant difference between the self-designed (mean Ct improvement: 7.3 ± 0.27 Ct values; PreAmp uniformity related to CDKN1B: -0.42 ± 0.29, and related to HPRT-CCM: -0.17 ± 0.30, respectively) and the inventoried ABI gene assays (mean Ct improvement: 7.20 ± 0.36; PreAmp uniformity related to CDKN1B: -0.36 ± 0.36, and related to HPRT-CCM: -0.06 ± 0.36, respectively; p each >0.05). Considering the threshold Ct for low expression levels published by Canales et al [18], 48/337 (14%) of the target genes showed Ct values ≥ 35 in direct real-time RT-PCR, whereas Ct values for HPRT-CCM demonstrated <28, and for CDKN1B < 26 in each case of the 4 investigated EMBs. There was no association between the PreAmp uniformity related to CDKN1B of Ct values below (-0.33 ± 0.6) or above (-0.4 ± 0.34) the threshold Ct for low expression levels (35). The same was true for the PreAmp uniformity values related to HPRT-CCM (Ct < 35: -0.03 ± 0.6; Ct ≥ 35: -0.1 ± 0.35). The inter-assay variance of Ct improvement for the investigated gene assays preamplified in each 4 different EMBs was below 0.5 Ct values (Figure 4), and the resulting mean CV was 1.89 ± 0.48% (range: 0.75 to 3.39), including the CD56 gene assay, which did not perform differently compared to the remaining gene assays in this respect (Figure 5). ABI inventoried gene assays demonstrated a higher inter-assay CV (1.99 ± 0.46%) compared with the self-designed gene assays (1.74 ± 0.46%; p = 0.0135). Noticeably, there was no traceable gene expression in some of the investigated each EMBs per gene group with respect to TRBV7, 10, 14, 15, 16, 24, 25, 27, 28, IL2, IL5, IL10, IFNb and APN neither in the direct nor in the PreAmp real-time RT-PCR (Figure 4).

**Figure 6:**
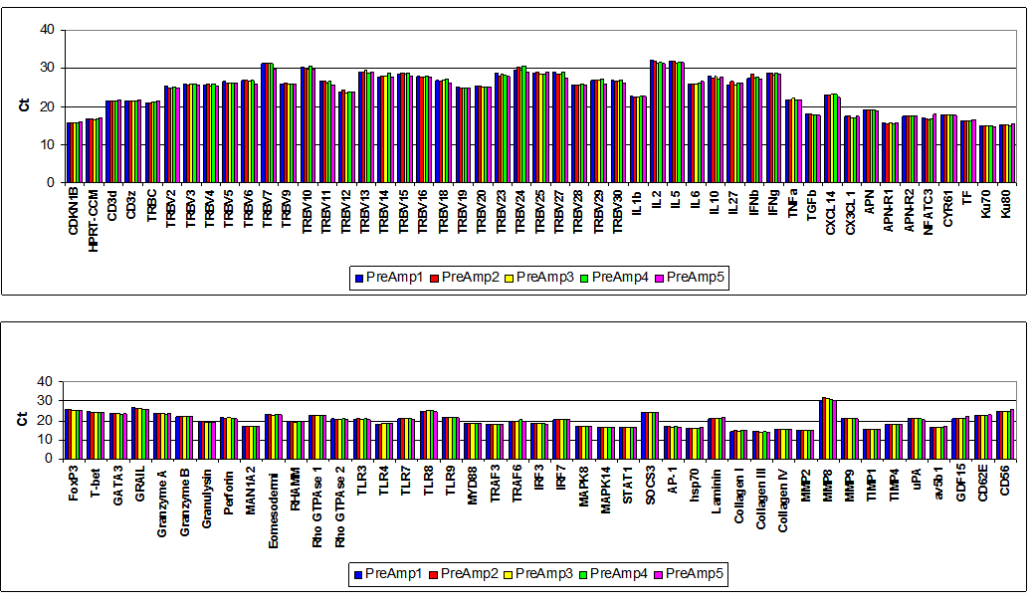
**Intra-assay variance in EMBs using T-PreAmp**. Ct values resulting from 5 different T-PreAmp reactions from cDNA aliquots derived from one EMB illustrated in 5 different colors. Taqman^® ^ABI inventoried gene assays are indicated by red bars, self-designed gene assays designed by coworkers of the Charité are indicated by blue bars.

**Figure 7:**
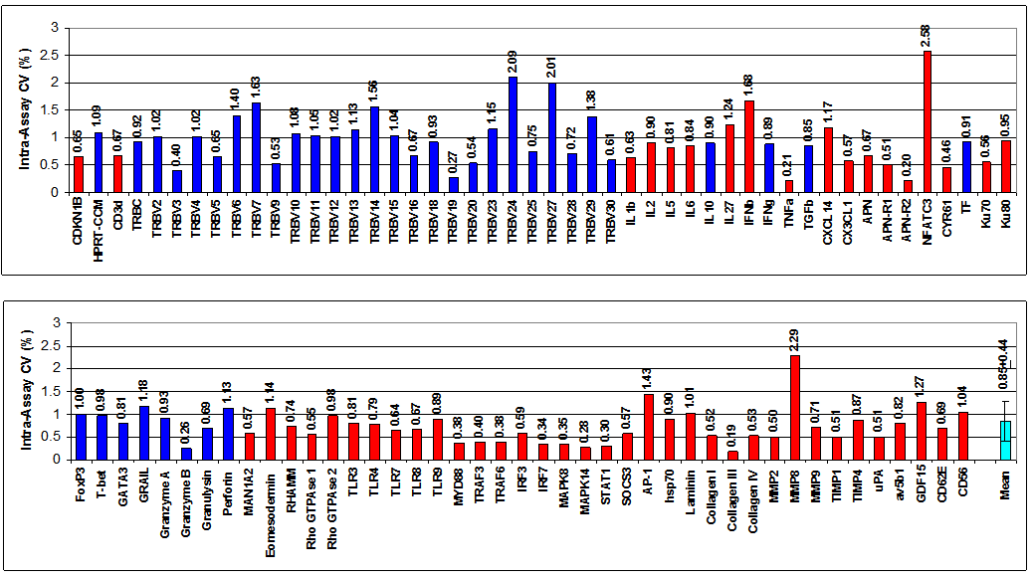
**Intra-assay CVs in EMBs using T-PreAmp**. Intra-assay CVs resulting from 5 different T-PreAmp reactions from cDNA aliquots derived from one EMB illustrated. Taqman^® ^ABI inventoried gene assays are indicated by red bars, self-designed gene assays designed by coworkers of the Charité are indicated by blue bars. The turquoise bar indicates the mean ± SD from all investigated gene assays.

The 5 different PreAmp procedures from the cDNA from 1 EMB resulted in minor differences of Ct values (Figure 6). The mean intra-assay CV was 0.85 ± 0.45% (range: 0.19 to 2.58), including CD56, which did not perform differently compared to the remaining gene assays with regard to the intra-assay CV (Figure 7). ABI inventoried gene assays demonstrated a lower intra-assay CV (0.77 ± 0.45%) compared with the self-designed gene assays (0.98 ± 0.41%; p = 0.0274) (Figure 6).

### SSRT-PreAmp (sequence-specific reverse transcription followed by a multiplex PreAmp)

**Figure 8:**
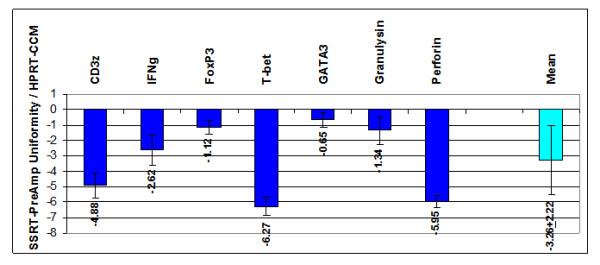
**PreAmp uniformity in EMBs using SSRT-PreAmp related to HPRT-CCM**. Values are displayed as means ± SD. The turquoise bar indicates the PreAmp uniformity mean ± SD of all investigated self-designed gene assays.

**Figure 9:**
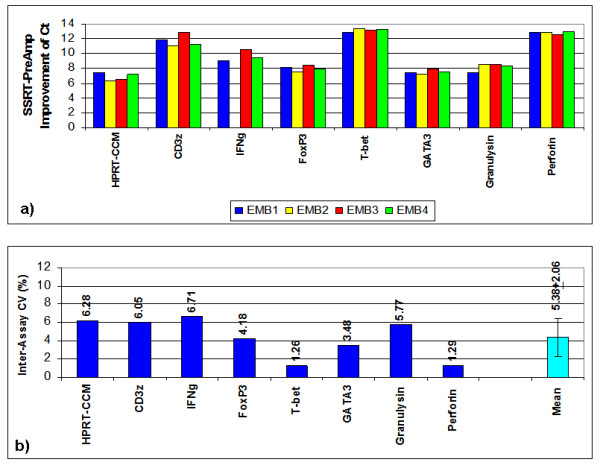
**Inter-assay variance and CV of Ct improvement in EMBs using SSRT-PreAmp**. a) CV of Ct improvement by T-PreAmp and b) inter-assay CVs for the investigated gene assays derived from 4 different EMBs (illustrated in 4 different colors) by SSRT-PreAmp. The turquoise bar indicates the mean ± SD of the investigated gene assays.

**Figure 10:**
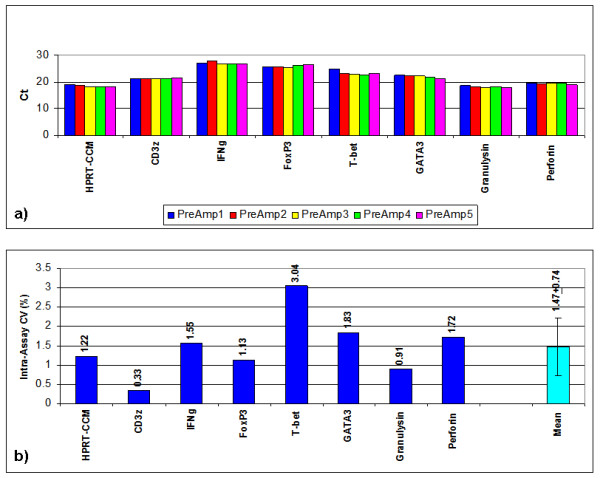
**Intra-assay variance and CVs in EMBs using SSRT-PreAmp**. a) Ct values resulting from 5 different T-PreAmp reactions and b) the resulting intra-assay CVs from cDNA aliquots derived from one EMB by SSRT-PreAmp. The turquoise bar in panel b indicates the mean ± SD from all investigated gene assays.

Employing SSRT-PreAmp, the investigated gene assays yielded a mean improvement of 9.76 ± 2.45 Ct values (range: 6.34 to 13.32). The mean Ct values obtained by direct real-time RT-PCR were 33.07 ± 4.27 (range: 24.57–31.19), and the SSRT-PreAmp Ct values were 23.31 ± 3.65 (range: 17.97–29.75; p < 0.0001 compared with the Ct values derived from the direct real-time RT-PCR), respectively. In one EMB, no traceable IFNg expression was observed neither by direct nor by SSRT-PreAmp real-time RT-PCR. Considering the threshold Ct for low expression levels published by Canales et al [18], 12/27 (44%) of the target genes showed Ct values ≥ 35 in direct real-time RT-PCR, whereas Ct values for HPRT-CCM demonstrated <29 in each case of the 4 investigated EMBs. The mean PreAmp uniformity of the investigated gene assays related to HPRT-CCM was -3.29 ± 2.40 (range: -6.98 to 0.05; Figure 8), which was significantly (p < 0.0001) lower compared with the T-PreAmp data. As in PBMCs, the gene assays for CD3z, IFNg, T-bet and Perforin performed below the PreAmp uniformity value of -1.5. The mean inter-assay CV using the SSRT-PreAmp technique was higher compared with T-PreAmp (5.38 ± 2.06; p = 0.01; Figure 9). There was no association between the PreAmp uniformity related to HPRT-CCM of target gene Ct values in direct real-time RT-PCR < 35 (-3.1 ± 2.49) or ≥ 35 (-3.51 ± 2.37). However, the SSRT-PreAmp yielded comparably low intra-assay CVs as obtained by T-PreAmp (Figure 10).

The real-time RT-PCR results from each 10 DCMi- and DCM-patients revealed differential expression with respect to 30 out of the 92 (32%) investigated target genes. These differential gene expressions were related to both HPRT-CCM and CDKN1B. Significantly different gene expressions related to HPRT-CCM are shown in Table 1. The data of all investigated target genes related to HPRT-CCM, including the non differentially expressed genes, are listed in the Additional file 1. Gene expression data related to CDKN1B are listed in the Additional file 2. Significantly increased gene expression was determined with respect to several T-cell related genes (CD3d, CD3z, TRBC, TRBV2, TRBV4, TRBV6, TRBV10, TRBV20, TRBV23, TRBV24, TRBV29, NFATC3 (nuclear factor of activated T-cells C3), interleukin 6 (IL6), tumor necrosis factor alpha (TNFa), the chemokine CX3CL1 (fractalkine), cysteine-rich angiogenic inducer 61 (CYR61), Th1 associated genes (T-bet and eomesodermin), genes expressed by cytotoxic T-lymphocytes (granzymes A and B, granulysin), β_1_-integrins (av5b1), the selectin CD62E (E-selectin) and growth-differentiation factor 15 (GDF15). The significantly down-regulated genes in EMBs from DCMi-patients were the chemokine CXCL14 (MIP-2g), adiponectin (APN) and tissue factor (TF).

**Table 1:** Differential gene expression results in EMBs from DCM and DCMi patients related to HPRT-CCM and CDKN1B using T-PreAmp

**Gene**	**DCM**	**DCMi**	**p value**
**CD3d**	0.014163 ± 0.008761	0.045395 ± 0.015252	<0.0001
**CD3z**	0.043139 ± 0.010119	0.060631 ± 0.007718	0.0023
**TRBC**	0.015027 ± 0.010150	0.027622 ± 0.010532	0.0098
**TRBV2**	0.001487 ± 0.001300	0.004503 ± 0.002881	0.0053
**TRBV4**	0.001501 ± 0.001432	0.005441 ± 0.003850	0.0047
**TRBV6**	0.002926 ± 0.002154	0.005478 ± 0.002562	0.0307
**TRBV10**	0.000186 ± 0.000127	0.000905 ± 0.000967	0.0245
**TRBV20**	0.003935 ± 0.003140	0.019818 ± 0.018318	0.0105
**TRBV23**	0.000408 ± 0.000269	0.006402 ± 0.008324	0.0270
**TRBV24**	0.000282 ± 0.000265	0.004754 ± 0.006232	0.0075
**TRBV29**	0.001302 ± 0.001168	0.002815 ± 0.00138	0.0192
**IL6**	0.002020 ± 0.001246	0.004133 ± 0.002452	0.0094
**TNFa**	0.028511 ± 0.014230	0.052024 ± 0.017001	0.0049
**CXCL14**	0.151853 ± 0.145437	0.054158 ± 0.052000	0.0299
**CX3CL1**	0.96874 ± 0.431717	1.39651 ± 0.790158	0.0245
**APN**	0.390377 ± 0.555952	0.035850 ± 0.058782	0.0092
**NFATC3**	1.29493 ± 0.479396	2.56855 ± 0.776956	0.0052
**CYR61**	0.85785 ± 0.47640	2.94840 ± 2.33843	<.0001
**TF**	3.59608 ± 1.01046	2.14158 ± 0.53169	0.0109
**T-bet**	0.014431 ± 0.005826	0.022267 ± 0.008127	0.0355
**Granzyme A**	0.014704 ± 0.006914	0.035028 ± 0.011726	0.0004
**Granzyme B**	0.062487 ± 0.022406	0.105532 ± 0.026082	0.0027
**Granulysin**	0.206285 ± 0.186560	0.532339 ± 0.327002	0.0183
**Eomesodermin**	0.014428 ± 0.007246	0.036024 ± 0.007900	0.0011
**av5b1**	1.04571 ± 0.283760	1.71316 ± 0.844310	0.0033
**GDF15**	0.020984 ± 0.013187	0.041715 ± 0.028283	0.0127
**CD62E**	0.002714 ± 0.001093	0.012877 ± 0.008890	0.0119

Significantly different gene expression (E; normalized to the housekeeping genes HPRT-CCM or CDKN1B) T-PreAmp real-time RT-PCR results in EMBs from DCM patients compared with EMBs from patients with immunohistologically confirmed DCMi (>7 CD3^+ ^infiltrates/mm^2^). The values are given as means ± SD, followed by the respective p values in significantly different comparisons.

### HPRT and CDKN1B are not regulated in DCM compared with donor hearts

**Figure 11:**
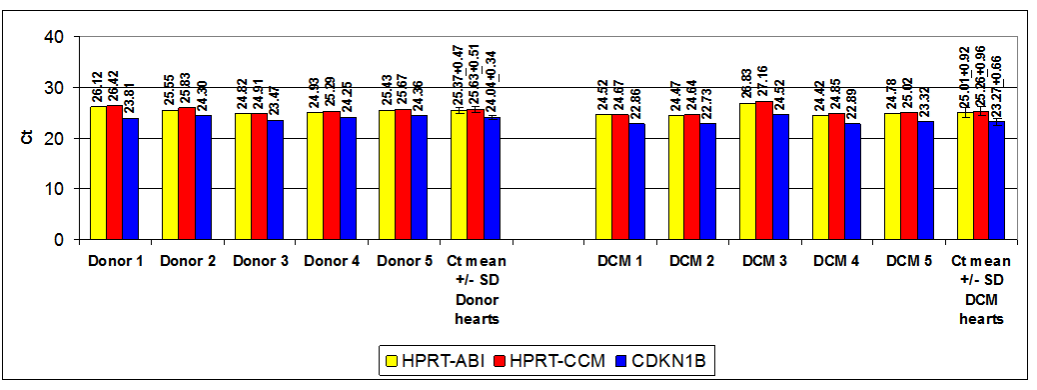
**HPRT-ABI, HPRT-CCM and CDKN1B in donor and DCM hearts**. Ct values of HPRT-ABI, HPRT-CCM and CDKN1B in each 5 donor hearts and 5 explanted DCM hearts (RNA subjected to cDNA synthesis adjusted to 50 ng/μl).

The value of HPRT as housekeeping gene is well acknowledged in various tissues. The close association between HPRT-CCM and CDKN1B Ct values as well as the equally significant differential expression of genes related to both HPRT-CCM and CDKN1B in EMBs from DCM versus DCMi patients infer a possible relevance of CDKN1B as a housekeeping gene. To address this issue, we determined the Ct values of HPRT (both HPRT-ABI and HPRT-CCM) and CDKN1B by direct real-time RT-PCR in each 5 explanted donor and terminally failing DCM hearts, from which enough tissue amounts were accessible to adjust RNA to 50 ng/μl before cDNA synthesis. As stated above for PBMCs, HPRT-ABI and HPRT-CCM demonstrated a difference around 0.3 Ct values. There was no statistical difference of Ct values of HPRT-ABI, HPRT-CCM and CDKN1B in donor and DCM hearts (Figure 11).

## Discussion

It is widely accepted that conventional histological diagnostics are not sufficient to elucidate the complex processes critically involved in the pathogenesis of DCMi, that they clearly lack prognostic impact, and that they are not sufficient to select DCM/DCMi patients who will likely benefit from immunomodulatory treatment [9,19]. Gene expression investigations are promising approaches for a profound understanding of the pathogenesis of DCM/DCMi, the prognostic impact of gene expression profiles in the natural course of the disease as well as under immunomodulatory treatment [4,11-13,16]. However, the amount of tissue and RNA/cDNA available from EMBs is limited, which restricts the number of analyzable target genes per EMB. PreAmp procedures may enhance sensitivity of real-time RT-PCR especially for low abundance expressed genes, and can establish substantially higher cDNA amounts, which can then expand the number of the analyzable target genes from a limited cDNA. However, maintenance of gene expression profiles as well as a broad applicability to many target genes with a feasible additional effort is a further intriguing aspect of PreAmp procedures.

The T-PreAmp technique enables a relatively simple workflow, which basically consists of setting up the primer/gene assay pool and T-PreAmp of cDNA over 14 cycles in a special master mix, maintaining robust PreAmp conditions for several gene assays, which results in mostly stable PreAmp uniformity values. Identical primers are used for both the T-PreAmp reaction and the succeeding real-time RT-PCR. This is substantially easier to accomplish compared with the SSRT-PreAmp, which requires design of specific primers for both the SSRT and the following preamplification step [17].

According to the manufacturer's specifications, up to 100 gene assays can be preamplified simultaneously in each T-PreAmp reaction, which is confirmed by our data on the simultaneously preamplified 92 gene assays both in PBMCs' and EMBs' cDNA. A further advantage of this technique is that out of 60 μl cDNA obtained from the herein applied RNA extraction and cDNA synthesis methods for EMBs, 8 different PreAmp reactions are feasible from one single EMB, which would hypothetically enable gene expression analysis of 784 different target genes (minus one housekeeping gene such as HPRT, and CDKN1B as the suggested reference gene for PreAmp uniformity). This is a substantially higher number of quantifiable target genes compared with the direct conventional real-time PCR, by which a maximum of 30 different target genes would be applicable in duplicate real-time RT-PCR analyses and 1 μl cDNA used in each reaction, and also compared with the SSRT-PreAmp procedure, which equally does not grant the possibility of expanding the maximum possible real-time RT-PCR analyses out of a limited amount of cDNA. This fact implies a further advantage of a negligible sampling error for this high putative number of real-time RT-PCR analyses carried out from one EMB, as opposed to the situation in which gene expression analyses would have to be split into different groups of target genes on cDNA from several EMBs from patients, given the limited RNA/cDNA amount available from each EMB for direct real-time RT-PCR analyses. Noticeably, sampling error is a critical issue for the histological assessment of myocardial inflammation [20].

The T-PreAmp technique with 14 PreAmp cycles results in a substantial increase of sensitivity of the real-time RT-PCR by around 7 Ct values, with PreAmp uniformity values ranging between -1.5 to +1.5 both in PBMCs' and EMBs' cDNA (except for the gene assays HPRT-ABI and CD56). The low intra- and interassay CVs in EMBs cDNA (<4%, including CD56) imply a high precision and repeatability of the T-PreAmp procedure. Furthermore, our data show that the PreAmp uniformity is maintained over a broad range of Ct values in direct real-time PCR, including low abundance Ct ranges (Ct ≥ 35 in direct real-time RT-PCR) [18]. This applies to both Taqman^® ^ABI inventoried gene assays as well as to self-designed gene assays adhering to ABI recommendations. As shown herein, only the forward/reverse primers of self-designed gene assays need to be included in the PreAmp Master Mix reaction, and do not perform significantly different compared with the ABI inventoried Taqman^® ^gene assays regarding the PreAmp uniformity, of which both primers and probes have to be pooled in the T-PreAmp reaction. Moreover, we determined that the PreAmp uniformity is also maintained with regard to gene assays using common reverse primers and common probes but diverse forward primers (TRBV primers) as well as to wobbled primer designs (TRBV5, 6 and 7 forward primers). These numerous advantages cannot be met by the SSRT-PreAmp procedure. First, this technique is not feasible for ABI inventoried Taqman^® ^gene expression assays, since the primer sequences of these assays are not accessible, and therefore primer design for the SSRT-PreAmp steps is not possible. Secondly, SSRT-PreAmp results on both PBMCs' and EMBs' cDNA demonstrated highly diverging PreAmp uniformity, with values below the suggested range of -1.5 to 1.5 with regard to CD3z, IFNg, T-bet and Perforin, albeit the same gene assays performed within the suggested PreAmp uniformity range of -1.5 to 1.5 using the Taqman^® ^PreAmp Master Mix. However, our data show that both PreAmp procedures perform without significantly different PreAmp uniformity values over a broad range of Ct values in direct real-time PCR, including low abundance Ct ranges (Ct ≥ 35 in direct real-time RT-PCR) [18], which implies a potential application in preclinical diagnostics especially for the T-PreAmp procedure due to its numerous advantages (i.e. robust PreAmp uniformity values for most gene assays, easier to use, expansion of possible real-time PCR analyses out of limited RNA/cDNA). One further important finding is the lack of traceable expression in both cDNA and T-PreAmp cDNA for target genes and samples, in which no detectable Ct values were obtained neither by direct real-time RT-PCR in cDNA from both PBMCs and EMBs. This infers that both T- and SSRT-PreAmp techniques yield reliable results and do not produce erroneous values.

As a limitation of our study, the comparison of PreAmp uniformity values of the investigated gene assays between T-PreAmp and SSRT-PreAmp was based on HPRT-CCM only, and not on CDKN1B, since the latter Taqman^® ^ABI inventoried gene assay is not applicable to SSRT-PreAmp. However, T-PreAmp data on HPRT-CCM indicate that this gene assay can be equally used as referral gene assay for the calculation of the PreAmp uniformity. This infers that not the design characteristics of these gene assays (i.e. PCR efficiency), but rather the PreAmp procedure per se is the decisively important issue leading to the observed discrepancies of PreAmp uniformity values. It is possible that interactions of the primers of the different gene assays during the SSRT-PreAmp procedure may lead to the observed bias in PreAmp uniformity. This is in line with our observations on the more pronounced distortion of the PreAmp uniformity values when adding more gene assays into the SSRT-PreAmp reactions (data not shown). Due to the higher complexity of the SSRT-PreAmp procedure and the expected higher chance of primer interactions during PreAmp, we did not investigate the PreAmp characteristics of the gene assays using common reverse primers/probes and diverse forward primers, nor wobbled primer designs (TRBV primers), with the SSRT-PreAmp procedure. Nested PCR techniques have a higher specificity compared with non-nested PCR [21], and real-time RT-PCR is known to have a higher specificity compared with nested PCR [22]. Thus, the advantage of the SSRT-PreAmp approach might be the higher specificity due to the nested primer design. Since the recipe of the T-PreAmp Master Mix^® ^is not accessible, we cannot speculate on the decisively important factors responsible for the observed disparity of the PreAmp uniformity of the T-PreAmp and the SSRT-PreAmp, so far. Possibly, the SSRT before the multiplex nested PreAmp may lead to the high diversity of PreAmp uniformity of the SSRT-PreAmp procedure, and this distortion is more pronounced regarding the gene assays CD3z, IFNg, T-bet and Perforin. Nonetheless, the equally low intra-assay CVs using both SSRT-PreAmp and T-PreAmp indicate that the SSRT-PreAmp is an equally reproducible approach, albeit potentially leading to more pronounced skewing of the calculation of relative gene expressions. However, the higher inter-assay CVs obtained by SSRT-PreAmp, although still <10%, indicate that the T-PreAmp technique leads to a higher precision for the direct comparison in the relative quantification of gene expression analyses between samples.

Our data show that the PreAmp uniformity of the T-PreAmp technique depends on the respective gene assay both in PBMCs' and EMBs' cDNA, and implies that gene assays should be tested for PreAmp uniformity before setting up serial T-PreAmp investigations. The two gene assays, which did not perform comparably to the suggested T-PreAmp uniformity reference gene assay CDKN1B, were HPRT-ABI and CD56 (both Taqman^® ^ABI inventoried gene assays). As a consequence, calculation of expression profiles of all target genes would be substantially altered, if HPRT-ABI would be used as a housekeeping gene assay in T-PreAmp real-time RT-PCR analyses, which emphasizes the value of PreAmp uniformity testing referring especially to a possible housekeeping gene candidate. In line with our observations, Denning et al. previously identified that the GAPDH Taqman^® ^ABI inventoried gene assay does not maintain the suggested PreAmp uniformity values. Therefore, these authors also normalized gene expression to CDKN1B, as well [23]. Nonetheless, as shown for CD56, the low inter- and intra-assay variations of the PreAmp technique (CVs <4%) warrants a high reproducibility of the PreAmp real-time RT-PCR results, even for the gene assays identified to show substantially altered PreAmp uniformity values out of the suggested range between -1.5 and +1.5. However, in the context of the relatively robust PreAmp uniformity performance of the T-PreAmp technique (especially compared to the SSRT-PreAmp procedure), one should also consider the PCR efficiency of the respective gene assays, since the "true" range of PreAmp uniformity hypothetically rises with decreasing PCR efficiency. Furthermore, the impact of contaminating DNA can be theoretically significant in such low-level expression levels of the target transcripts. The intron spanning primer design avoiding co-amplification of genomic DNA of both the self-designed and of the Taqman^® ^ABI inventoried gene assays, however, and the missing cross reactivity with genomic DNA tested in DNA from PBMCs largely excludes an impact of this theoretically problematic issue in our investigations. Our investigations on possible causes for disproportionate T-PreAmp uniformities, although not elucidating the concrete reason, reveal that the T-PreAmp performance does not depend on the amplicon length and on the PCR efficiency of the respective gene assays. Denning et al. deduced from their investigations on GAPDH (122 bp) and 12 further gene assays with shorter amplicon lengths, that the amplicon length might be crucially important for T-PreAmp uniformity [23]. However, our data cannot confirm this hypothesis, especially since the TRBV19 and TRBV29 gene assays yielding amplicons around 260 bp and 320 bp (depending on the respective diversity and joining regions) resulted in PreAmp uniformities in the range between -1.5 and +1.5, performing comparably to further self-designed gene assays (i.e. HPRT-CCM: 101 bp; CD3z: 108 bp; TRBC: 151 bp), and Taqman^® ^ABI inventoried gene assays (i.e. CD3d: 92 bp; NFATC3: 74 bp; av5b1: 75 bp). There was no evidence for an impact of the PCR efficiency of these gene assays on the PreAmp uniformity. In contrast, the Taqman^® ^ABI inventoried gene assays HPRT-ABI (100 bp) and CD56 (61 bp) showed substantially lower improvement of Ct values under the same conditions. Noticeably, this distortion of PreAmp uniformity was stable at the serial cDNA dilutions regarding HPRT-ABI, whereas the CD56 gene assay showed an increasing PreAmp uniformity with decreasing PCR efficiency at serial cDNA dilutions. We therefore conclude that distortion of T-PreAmp uniformity is gene assay specific and not a general problem of the T-PreAmp technique. These findings add clarity, however, are troublesome at the same time, since the concrete reason for disproportionate T-PreAmp performance of certain gene assays still remains unclear. Nonetheless, these data again highlight the importance of PreAmp uniformity pretests before setting up serial investigations using the T-PreAmp technique. To gather, compare and discuss the experiences especially on the obvious distortion of T-PreAmp uniformity with regard to particular gene expression assays, we propose the constitution of a central open access forum. In light of the possible impact of primer interactions during the T-PreAmp reaction, of RNA quality and DNA contamination, this forum should not only refer to the T-PreAmp performance of single gene assays, but also incorporate the precise gene assay mix in the respective T-PreAmp reaction, the applied RNA extraction technique, RNA quality and DNA contamination issues. Hopefully, the decisively important design characteristics of gene assays leading to a substantial deviation of the T-PreAmp uniformity can be identified, eventually. These insights might lead to additional rules of gene assay design, by which the T-PreAmp uniformity can be predicted.

Several gene expression analyses have been reported comparing heart failure to non-failing hearts, or different cardiomyopathy entities. In this preliminary analysis, we compared EMBs from 10 DCM patients with 10 patients with immunohistologically confirmed DCMi. Both patient groups had comparably depressed LVEF, which alleviates a possible bias of secondary general heart-failure associated mechanisms. Our preliminary data from these T-PreAmp real-time RT-PCR analyses revealed differential gene expression with respect to 27/90 (30%) of the investigated target genes. In brief, these investigations confirmed that the immunohistologically diagnosed DCMi is accompanied by a significantly increased expression of T-cell related genes (CD3d, CD3z, TRBC and NFATC3). Up-regulation of distinct TRBV families (TRBV2, 4, 6, 10, 20, 23, 24 and 29) in EMBs from DCMi patients indicates a selective recruitment and/or expansion, and therefore restriction of TRBV usage of the T-cell infiltrates in DCMi [24]. The increased expression of IL6, TNFa and CX3CL1 infer that T-cell infiltration in DCMi is paralleled by differential increment of these cytokines, whereas a further chemokine, CXCL14, was confirmed to be downregulated in DCMi patients as recently reported for DCMi patients with slightly impaired LVEF by microarray analyses [15]. Furthermore, in line with these microarray findings, our investigations confirmed a down-regulation of APN, while its receptors APN-R1 and APN-R2 were not differentially expressed, and an up-regulation of CYR61 [15]. The results on markers of T-cell activation reveal a yet not recognized potential role of Granzymes A and B, Granulysin, as well as T-bet and eomesodermin as markers for cytotoxic and Th_1_-polarized T-cells, respectively, in DCMi. In contrast, markers for Th_2_-polarized T-cells, anergic and regulatory T-cells (IL4, IL5, IL10, TGFb, GATA3, GRAIL, FoxP3) were not increased in EMBs from patients with immunohistologically confirmed DCMi, which might indicate a missing counter-balance of the T-cellular response in DCMi. The lack of differential expression of Toll-like receptors (TLRs) and signaling factors involved in TLR downstream pathways do not favor the hypothesis of a paramount role of TLRs in DCMi, so far. Our analyses furthermore confirmed downregulation of intramyocardial TF expression in DCMi versus DCM patients [25]. Whilst altered expression of several components of the extracellular matrix is well known in DCM and further non-DCM cardiomyopathies [14,26], no direct link to inflammation or cardiotropic viral infection has been described, so far. Our preliminary data do not show any differential expression of collagen types I, III and IV, MMPs 2, 8, 9, TIMPs 1, 4 and uPA in EMBs from DCMi versus DCM patients. Increased expression of β_1_-integrins and further adhesion molecules such as CD62E have been confirmed by immunohistological analyses [1], which is in line with our data on av5b1 and CD62E. GDF15 is a newly recognized marker of heart failure, and our data implicate an additional role of DCMi for increased GDF15 expression [27]. In summary, these preliminary analyses confirm that the T-PreAmp procedure, in addition to its technical applicability to EMBs, thereby expanding the number of analyzable target genes in EMBs investigations, is capable of revealing significantly different gene expression profiles in DCMi versus DCM patients. Albeit numerous reports have confirmed close associations between RNA and protein gene expression, a discrepancy between these two levels of gene expression was elucidated in some investigations, i.e. due to posttranslational modification [28,29]. We show here a significant association between expression of T-cellular markers (CD3d, CD3z, TRBC, NFATC3) and DCMi as determined by immunohistologically quantified CD3^+ ^infiltrates. Although it would be beyond the scope of this methodology paper, significantly different gene expression analyses as determined by PreAmp real-time PCR should be ideally confirmed by protein expression analyses in future investigations.

Further investigations on larger patient cohorts are warranted to explore associations with the various cardiotropic viruses [2,30], the impact of LVEF, the acuity of the disease (i.e. patients presenting with acute myocarditis versus DCM), the prognostic role of gene expression for the diverse natural course of the disease (improvement versus further deterioration under heart failure medication) [3], as well as the changes of gene expression in DCMi patients under immunomodulatory treatment modalities (i.e. immunosuppression, immunoadsorption, antiviral treatment; [4,11-13,16]). The application of the T-PreAmp procedure might be especially useful in light of the new EMB guidelines [31]. Moreover, these promising results of T-PreAmp real-time RT-PCR in EMBs might be stimulating for researchers analyzing biopsies or comparably small sized tissue samples from other organs, and for PreAmp real-time RT-PCR analyses from limited cell amounts such as those obtained by laser capture microdissection [23,32].

Finally, we here provide first evidence that CDKN1B, in addition to its function as the suggested reference gene for the PreAmp uniformity, can be used as a relevant housekeeping gene for the relative quantification of gene expression in real-time RT-PCR in myocardial tissues, equivalent to HPRT. Whereas the tumor suppressor gene function of CDKN1B is well understood in other tissues and pathologic conditions [33,34], the physiological role and expression pattern of CDKN1B in myocardial tissues has not been investigated, yet. Congruent with our observations, Denning et al. have previously confirmed that CDKN1B can also serve as housekeeping gene in thyroid tissues [23].

## Conclusion

In comparison to the SSRT-PreAmp, T-PreAmp enables a relatively simple workflow, and results in a robust PreAmp of multiple target genes (at least 92 gene assays as tested here) by a mean Ct improvement around 7 cycles, and in a lower inter-assay variance in RNA derived from EMBs. Preliminary analyses comparing EMBs from DCM and DCMi patients, revealing differential regulation regarding 30% of the investigated genes, confirm that T-PreAmp is a suitable tool to perform gene expression analyses in EMBs, expanding gene expression investigations with the limited RNA/cDNA amounts derived from EMBs. CDKN1B, in addition to its function as a reference gene for the calculation of PreAmp uniformity, might serve as a suitable housekeeping gene for real-time RT-PCR analyses of myocardial tissues.

## Methods

### Patients and sample preparation from peripheral blood monocytes (PBMCs) and myocardial tissues

Peripheral blood mononuclear cells (PBMCs) were separated from peripheral blood obtained from 5 adult healthy donors (each 3 ml) in Vacutainer^® ^EDTA tubes (BD Biosciences, Heidelberg, Germany) within 10 minutes after blood drawing using the LSM 1077 Lymphocyte Separation Medium (PAA, Pasching, Germany) density gradient. PBMCs were lysed in RLT^® ^buffer (Qiagen, Hilden, Germany), and total RNA was gained using the Qiagen spin column system according to the manufacturer's instructions. RNA from each individual was adjusted to 25 ng/μl. After reverse transcription (RT), cDNAs were diluted at 1:50, which resulted in comparable direct real-time RT-PCR Ct values as cDNA from EMBs. PBMCs' cDNA from these 5 subjects were pooled to minimize missing expressions especially for cytokines. RNA concentrations of both PBMCs and myocardial tissues were quantified using the Nanodrop^® ^ND-1000 spectrophotometer (Agilent Technologies, Böblingen, Germany).

EMBs from n = 36 DCM patients (8/22% females; age: 38.2 ± 12.7 years; LVEF: 37.7 ± 17.4%) were used for the determination of the mean improvement of Ct values and of the PreAmp uniformity related to CDKN1B and HPRT-CCM as well as the inter-assay variance of PreAmp using the T-PreAmp technique. Due to limited amounts of cDNA, groups of each 9 or 10 target gene assays in addition to CDKN1B and HPRT-CCM were determined in 4 EMBs per gene assay group. For the SSRT-PreAmp investigations, EMBs from 4 further DCM patients (all males, age: 41.2 ± 5.6 years, LVEF: 35.1 ± 9.1%) were used. Intra-assay variance was determined in 5 aliquots of cDNA from each one EMB for both PreAmp techniques, in which direct real-time RT-PCR showed expression of all investigated gene assays.

To determine preliminary data on differential gene expression by real-time PCR using the T-PreAmp technique, we selected EMBs from 20 patients presenting with DCM (LVEF: 25.1 ± 7.6%; age: 49.6 ± 13.8 years, males/females: 16/4). In ten of these patients, DCMi was confirmed by routine EMBs analyses (CD3^+ ^infiltrates: 14.8 ± 5.1/mm^2^; Parvovirus B19 in n = 6, human herpes virus type 6 in 3, and one case with co-infection with both viruses; [1,2]), whereas the EMBs of the remaining 10 patients did not show any immunohistological characteristics of increased T-cell infiltration (CD3^+^: 3.2 ± 2.3/mm^2^), and no cardiotropic viral infection.

Total RNA was isolated from EMBs (3–6 mg tissue) using TRIZOL^® ^(Invitrogen, Karlsruhe, Germany) and was purified using the ChargeSwitch^® ^Total RNA Cell Kit (Invitrogen, Karlsruhe, Germany). Mean RNA concentrations of the EMBs were 26.7 ± 13.2 ng/μl.

To ascertain whether CDKN1B and HPRT are regulated in myocardial tissues, we determined the Ct values for both genes in 5 snap frozen myocardial samples from donor hearts and from 5 explanted DCM hearts (all males; age: 45.6 ± 6.7 years; LVEF ≤ 25%) adjusted at 50 ng/μl RNA before retrotranscription.

Reverse transcription to cDNA from RNA extracted from both PBMCs and myocardial tissues was carried out using the high capacity archive kit (Applied Biosystems/ABI, Darmstadt, Germany) according to the manufacturer's instructions. 60 μl of PBMCs' RNA and 30 μl of RNA from EMBs were transcribed to a final volume of 120 μl and 60 μl, respectively.

These investigations were approved by the local ethics committee at the Charité – Universitätsmedizin Berlin in the framework of the Sonderforschungsbereich TR19 in compliance with the Helsinki Declaration. Every patient gave informed consent.

### Design of primers and probes and Taqman^® ^ABI inventoried gene assays

For the gene assay design of the T-cell receptor Vβ variable region (TRBV) and TRB constant region (TRBC) primers and probes, we used the gene sequences published at the Immunogenetics Database [35]. Primers and probes were designed using the Primer Express Software (version 2.0; ABI, Darmstadt, Germany) according to ABI recommendations. Sequence homologies between the various functional TRBVs were excluded by multiple alignment using ClustalX Software (version 1.83; University of Strasbourg, France). We chose TRBV specific forward primers and one common reverse TRB primer on the constant TRB (TRBC) region and a probe on the TRBC region (encompassing both human TRBC1 and TRBC2). A further gene assay was designed on the constant region of the TRBC (encompassing both human TRBC1 and TRBC2). For TRBV5, 6, and 7, wobbled (WBL) primers were designed to encompass few base differences. TRBV and TRBC minor groove binder (MGB) probes were synthesized by Applied Biosystems (ABI; Darmstadt, Germany). Primers and probes for HPRT-CCM, IL-10, IFN-g, TGF-b, FoxP3, T-bet, GATA3, granzyme A and B, granulysin and perforin were designed using the Oligo 4.1 software (Molecular Biology Insights, Cascade USA) and published genomic sequences (National Center for Biotechnology Information/NCBI) according to ABI recommendations. The sequences of the primers are listed in Table 2, and of the fluorescence hybridization probes in Table 3, respectively. All probes used were FAM dye (carboxyfluorescein) labeled. The primers and probe for tissue factor (TF) has been published elsewhere [25]. All primers and probes (except for TRBV and TRBC MGB-probes) were synthesized by Metabion (Martinsried, Germany). The ABI inventoried TaqMan^® ^gene expression assays are listed in Table 4. All self-designed gene assays followed an intron spanning design according to ABI recommendations, and thus, as well as the applied Taqman^® ^ABI inventoried gene assays, did not show any cross reactivity with genomic DNA extracted from PBMCs.

**Table 2:** Sequences of primers and probes of self-designed real-time RT-PCR gene assays

**Gene name**	**5' sense 3' primer sequence**	**5' antisense 3' primer sequence**
TRBC	TCCGCTGTCAAGTCCAGTTCTA	GACAGGACCCCTTGCTGGTA
TRBV common antisense primer		CTGCTTCTGATGGCTCAAACA
TRBV2	ACTCTGAAGATCCGGTCCACAA	
TRBV3	ATCAATTCCCTGGAGCTTGGT	
TRBV4	CCTGAATGCCCCAACAGC	
TRBV5	GCTCTGAGCTGAATGTGAACGC	
TRBV5WBL	GCTCTGAGATGAATGTGAGTGC	
TRBV6	CACTGACAAAGGAGAAGTCCC	
TRBV6WBL6-2	AACTGCCAAAGGAGAGGTCCC	
TRBV6WBL6-4	CACTGGCAAAGGAGAAGTCCC	
TRBV7	CTCTCAGGTGTGATCCAATTTCG	
TRBV7WBL7-2;7-3	AGCTCAGGTGTGATCCAATTTCA	
TRBV7WBL7-9	CTTTCAGGTGTGATCCAATTTCT	
TRBV9	CACAACAGTTCCCTGACTTGCA	
TRBV10	CATGGGCTGAGGCTGATC	
TRBV11	CTGCAGAGAGGCTCAAAGGAGTAG	
TRBV12	AGAACCCAGGGACTCAGCTGT	
TRBV13	GAACTGAACATGAGCTCCTTGGA	
TRBV14	CTGAAAGGACTGGAGGGACGTAT	
TRBV15	CAGGAGGCCGAACACTTCTTT	
TRBV16	GCCTCCCAAATTCACCCTGTA	
TRBV18	CCAGCATCCTGAGGATCCA	
TRBV19	ACTGTGACATCGGCCCAAA	
TRBV20	AACCATGCAAGCCTGACCTT	
TRBV23	CCCTGCAGCCTGGCAAT	
TRBV24	GCTAAATTCTCCCTGTCCCTAGAGT	
TRBV25	TTCCCCTGACCCTGGAGTCT	
TRBV27	GGCTTAAGGCAGATCTACTATTCAATG	
TRBV28	GCCAGCACCAACCAGACAT	
TRBV29	AGCCGCCCAAACCTAACATT	
TRBV30	CGGCAGTTCATCCTGAGTTCT	
HPRT-CCM	AGTCTGGCTTATATCCAACACTTCG	GACTTTGCTTTCCTTGGTCAGG
IL10	CAAGTTGTCCAGCTGCTTCAT	TTGGCGAAAGCAGCTCCTC
IFNγ	CAGGTCATTCAGATGTAGCGGATAA	AGGAGACAATTTGGCTCTGCATT
TGFβ	CCCTGCCCCTACATTGGAG	CCGGTTATGCTGGTTGTACA
FoxP3	TGGCAAACGGAGTCTGCAA	TCTCATCCAAGAGGTGATCTGCTT
T-bet	CAACACAGGAGGCGCACTGG	CCCCCTTGTTGTTTGTGAGCT
GATA3	CCTCATTAAGCCCAAGCGAAG	TTGGCATTTCCTCCAGAGT
GRAIL	GAAGCAGAGGCAATTAAAGGCAG	CATCAGGGCCAATTTCCTTGT
Granzyme A	CCCTGTGATTGGAATGAATATGGT	ACACCCTCGCACAACAAAGG
Granzyme B	GCGAATCTGACTTACGCCATTATT	CAAGAGGGCCTCCAGAGTCC
Granulysin	AAAACTGAAGAAGATGGTGGATAAGC	ATCGTGACCTCCCCGTCCTA
Perforin	GGACCAGTACAGCTTCAGCACTG	AGTCAGGGTGCAGCGGG

Sequences of primers of self-designed real-time RT-PCR gene assays. For the different TRBV forward primers, one common reverse primer and one common MGB hybridization probe was designed. Wobbled (WBL) forward primers were used for TRBV5, 6 and 7.

**Table 3:** Sequences of fluorescence hybridization probes

**Gene name**	**Sequence**
TRBC	ACGAGTGGACCCAGGATAGGGCCAA
TRBV common probe	CACCCGAGGTCGCT
HPRT-CCM	TTTCACCAGCAAGCTTGCGACCTTGA
IL10	TTACAGGACCAGAGGGACAAGACATGACTGTG
IFNγ	TTTCTGTCACTCTCCTCTTTCCAATTCTTCAAA
TGFβ	CACGCAGTACAGCAAGGTCCTGGCC
FoxP3	AGCCGGGAGAGTTTCTCAAGCACTGC
T-bet	CACCTGTTGTGGTCCAAGTTTAATCAGCACC
GATA3	TCCTGTGCGAACTGTCAGACCACCAC
GRAIL	GGAAGGCTTCAACTACGCACACTGAAACAAG
Granzyme A	CTTCCAGAATCTCCAATTGCGCGAGTCTCTTC
Granzyme B	CCCACGCACAACTCAATGGTACTGTCG
Granulysin	ACCCAGAGAAGTGTTTCCAATGCTGCGAC
Perforin	TGCCGCTTCTACAGTTTCCATGTGGTACAC

Sequences of fluorescence hybridization probes of self-designed real-time RT-PCR gene assays. For the different TRBV forward primers, one common MGB hybridization probe was used.

**Table 4:** ABI inventoried Taqman^® ^gene expression assays

**Gene name**	**ABI ID number**
CDKN1B	Hs00153277_m1
HPRT-ABI	Hs99999909_m1
CD3D	Hs00174158_m1
IL-1β	Hs00174097_m1
IL-2	Hs00174114_m1
IL-4	Hs00174122_m1
IL-5	Hs00174200_m1
IL-6	Hs00174131_m1
IL-17	Hs00174383_m1
IL-27	Hs00377366_m1
IFN-β	Hs01077958_s1
TNFα	Hs00174128_m1
CXCL14	Hs00171135_m1
CX3CL1	Hs00171086_m1
APN	Hs00605917_m1
APN-R1	Hs00360422_m1
APN-R2	Hs00226105_m1
NFATC3	Hs00190046_m1
CYR61	Hs00155479_m1
Ku70	Hs01922652_g1
Ku80	Hs00221707_m1
MAN1A2	Hs00198611_m1
Eomesodermin	Hs00172872_m1
RHAMM	Hs00234864_m1
RhoGTPAse1	Hs00205507_m1
RhoGTPAse2	Hs00183269_m1
TLR3	Hs00152933_m1
TLR4	Hs00370853_m1
TLR7	Hs00152971_m1
TLR8	Hs00152972_m1
TLR9	Hs00370913_s1
MYD88	Hs00182082_m1
TRAF3	Hs00377462_m1
TRAF6	Hs00270336_m1
IRF3	Hs00155574_m1
IRF7	Hs00242190_g1
MAPK8	Hs00177083_m1
MAPK14	Hs00176247_m1
STAT1	Hs00234829_m1
SOCS-3	Hs00269575_s1
AP-1	Hs00277190_s1
HSP70	Hs00382884_m1
Laminin	Hs00165042_m1
Collagen I	Hs00164004_m1
Collagen III	Hs00164103_m1
Collagen IV	Hs00266237_m1
MMP2	Hs00234422_m1
MMP8	Hs00233972_m1
MMP9	Hs00234579_m1
TIMP1	Hs00171558_m1
TIMP4	Hs00162784_m1
uPA	Hs00170182_m1
GDF15	Hs00171132_m1
CD62E	Hs00174057_m1
CD56	Hs00169851_m1
TIMP4	Hs00162784_m1

ABI inventoried Taqman^® ^gene expression assays and the respective ABI ID numbers.

### T-PreAmp procedure

**Figure 12:**
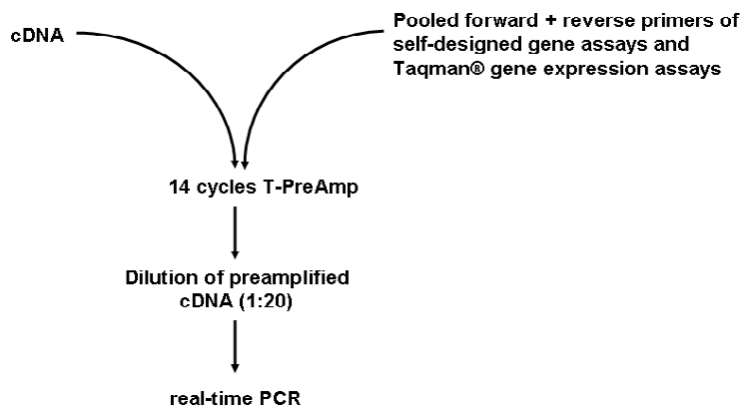
**T-PreAmp procedure**. Principle of the T-PreAmp technique: After standard reverse transcription, cDNA was preamplified (14 cycles) using a pool of all investigated self-designed gene assays (primers) and ABI inventoried Taqman^® ^gene expression assays (mixture of primers and fluorescence probes). The preamplified cDNA products were then diluted 1:20 and subjected to real-time PCR analyses.

The pooled TaqMan^® ^assays (including fluorescent probes) as well as the self-designed forward/reverse primers were diluted with 1 × Tris-EDTA (TE) buffer, so that each assay was at a final concentration of 0.2 fold in the PreAmp primer/assay pool according to the manufacturers' instructions. Since initial experiments comparing the volume of PreAmp reaction suggested by ABI (total PreAmp reaction volume: 50 μl) and the half of the volume of PreAmp reaction recommended by the manufacturer (25 μl PreAmp reaction volume) showed identical PreAmp results, we used the latter approach further on due to reasons of cost-effectiveness. The herein used PreAmp reaction conditions involved the amplification of 6.25 μl cDNA in a 25 μl reaction consisting of 12.5 μl TaqMan^® ^PreAmp Master Mix and 6.25 μl pooled primer/assay mix (0.2×, each assay). T-PreAmp of this primer/gene assay pool was carried for 14 cycles on a PTC-100 Programmable Thermal Controller (MJ Research, Inc., Watertown, Mass., USA) with the following program: denaturation at 95°C for 10 min and 14 cycles of amplification (15 sec at 95°C, 4 min at 60°C). The preamplified products were then diluted with Tris-EDTA-buffer at a ratio 1:20 (resulting volume 500 μl) and were used as templates for the real-time RT-PCR analysis (Figure 12).

### SSRT-PreAmp procedure

**Figure 13:**
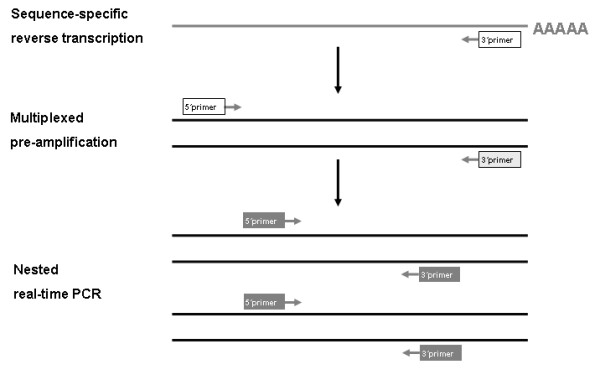
**SSRT-PreAmp procedure**. Principle of the SSRT-PreAmp technique: Isolated RNA was reverse transcribed to cDNA using a sequence specific 3'primer for each gene of interest (white box). Generated cDNA was preamplified (10 cycles) in a multiplex PCR by using the specific 3'primer of cDNA-synthesis and a specific 5'primer (light grey box). Next, products of the first PCR round were split into individual wells and amplified separately in real-time PCR applying nested amplification primers (dark grey boxes).

SSRT-PreAmp was applied to 8 self-designed gene assays (HPRT-CCM and 7 T-cell related gene assays: CD3z, IFNg, FoxP3, T-bet, GATA3, Granulysin and Perforin). Isolated RNA was reverse transcribed using a sequence-specific 3' primer instead of oligo dT primers (Table 5). This sequence specific reverse transcription was carried out simultaneously for every gene of interest (n = 8) in the same reaction. Afterwards, a first multiplex PCR consisting of 10 cycles was run, in which all genes were amplified simultaneously adding the amplification primers. Next, a real-time RT-PCR applying nested amplification primers and fluorogenic probes was performed amplifying each gene separately. cDNA was used in a first round of multiplex PCR amplification. An aliquot of 5 μl of the RT reaction was amplified in a final volume of 20 μl of PCR mix containing 50 mmol/l KCl, 10 mmol/l Tris-HCl at pH 8.3, 2 mmol/l MgCl2, 0.2 mmol/l dNTPs, 3 units of AmpliTaq Gold DNA Polymerase (ABI; Darmstadt, Germany), and 0.015 μmol/l of specific amplification primers for all investigated self-designed genes (Table 2). The 3' primers applied were the same as for cDNA synthesis. The cycle program consisted of one step of denaturation at 95°C for 10 min and 10 cycles of amplification (45 sec at 95°C, 60 sec at 62°C, 90 sec at 72°C). The SSRT-PreAmp steps are illustrated in Figure 13.

**Table 5:** Preamplification primers for SSRT-PreAmp

**Gene**	***5' sense 3' primer***	***5' antisense 3' primer***
**HPRT-CCM**	GATATAATTGACACTGGCAAAAC	ACAAACATGATTCAAATCCCTGA
**CD3z**	CCGATTACAGAGGCACAGAG	TCGTACTCCTCTCTTCGTCC
**IFNg**	TTTTGGGTTCTCTTGGCTGTTA	CTCTTTTGGATGCTCTGGTCA
**FoxP3**	CCCAGAGCTCCTACCCACT	TGGCACTCAGCTTCTCCTTC
**T-bet**	GACTGGAGGTGTCGGGGAA	CCACGTCCACAAACATCCTG
**GATA3**	TCACAAAATGAACGGACAGAAC	TAATATTGTGAAGCTTGTAGTAGA
**Granulysin**	AGGAGCTGGGCCGTGACTA	CTCTAGACTGATACCTCCTCA
**Perforin**	TGTGGCCGGCTCACACTCA	CGTAGTTGGAGATAAGCCTGA

Sequences of primers used for SSRT-PreAmp of the investigated 8 target genes.

### Real-time RT-PCR, PreAmp uniformity, intra- and inter-assay variance

The real-time RT-PCR reactions were performed in a final volume of 12.5 μl, containing 2 μl cDNA, 6.25 μl Master Mix (TaqMan™ Universal PCR Master Mix, No AmpErase^® ^UNG, ABI, Darmstadt, Germany), 0.21 μl probe (final concentration 0.05 μM) and 1.25 μl forward and reverse primers (final concentration 0.9 μM). The PCR reactions for TaqMan^® ^gene expression assays contained 2 μl cDNA, 6.25 μl Master Mix and 0.625 μl TaqMan^® ^gene expression assay. Reactions were made up to a final volume of 12.5 μl with sterile water. All experiments were performed in duplicate on an ABI 7900 HT Fast real-time PCR System (Applied Biosystems, Darmstadt, Germany). The real-time RT-PCR protocol was as following: Denaturation by a hot start at 95°C for 10 min, followed by 40 cycles of a two-step program (denaturation at 95°C for 15 sec and annealing/extension at 60°C for 1 min). Gene expression (E) was normalized to HPRT-CCM or CDKN1B applying the formula: 2^-ΔCt^.

PreAmp uniformity related to CDKN1B (reference gene suggested by ABI for the determination of PreAmp uniformity) and HPRT-CCM was calculated according to the following formula:

Δ*Ct PreAmp *= average *Ct target *× *[PreAmp] *- average *Ct CDKN1B or HPRT-CCM [PreAmp]*.

Similarly, the average Ct from non-PreAmp cDNA template were normalized to the average Ct of the uniformity reference gene according to the following formula:

Δ*Ct cDNA *= average *Ct target *× *[cDNA] *- average *Ct CDKN1B or HPRT-CCM [cDNA]*.

The ΔΔCt was determined by the difference of the two ΔCts:

ΔΔ*Ct *= Δ*Ct [PreAmp] *- Δ*Ct [cDNA]*.

A ΔΔCt close to zero indicates ideal PreAmp uniformity. ABI suggests a range between -1.5 to +1.5 PreAmp uniformity values as acceptable.

The coefficients of variation (CV) of the intra- and inter-assay variance were calculated according to the formula SD/meanx100.

To ascertain the possible impact of PCR efficiency on T-PreAmp uniformity, cDNA from the pooled PBMCs was diluted at 1:4, 1:16, 1:32, 1:64, 1:256, 1:512 and 1:1,024. PCR efficiency was calculated according to the formula: -1+10^(-1/slope)^. The data from direct and from T-PreAmp real-time PCR on candidate (both self-designed and Taqman^® ^ABI inventoried) gene assays with T-PreAmp uniformities ranging between -1.5 and +1.5 (HPRT-CCM, CD3d, CD3z, TRBC, TRBV19, TRBV29, NFATC3 and av5b1), as well as on the 2 gene assays showing T-PreAmp uniformities out of this range (HPRT-ABI and CD56) were compared.

### Statistical analysis

Statistical analysis was performed using JMP Statistical Discovery Software V5.1.2 (SAS Institute, Inc., Cary, NC, USA). Data are expressed as means ± standard deviation (SD). We compared ordinal with continuous data employing the student's t-test. Continuous data were compared using the Spearman ρ test. A probability value of p < 0.05 was considered statistically significant.

### Commonly used abbreviations

For the list of abbreviations commonly used in the manuscript see Additional File 3.

## Authors' contributions

MN was involved in the design and coordination the study, setting up the PreAmp protocol using the Taqman^® ^PreAmp Master Mix Kit, in the design of the TRBC/TRBV gene assays, in the statistical analysis, in the immunohistological EMBs analyses, and he wrote the manuscript. MR established and carried out the PreAmp procedure and the majority of the real-time RT-PCR analyses, and designed the TRBC/TRBV gene assays, and was involved in the statistical analysis and editing the manuscript. AB, KaKl, OL and KB were involved in RNA-extraction, retrotranscription, carrying out the PreAmp and real-time RT-PCR reactions, statistical analysis of the data, and they edited the manuscript. In addition, AB was involved in the design of the TRBC/TRBV gene assays. MH was involved in the overall design of study, in the design of the TRBC/TRBV gene assays, reviewed the data, and edited the manuscript. MP, WP, UK and HPS were involved in EMBs obtainment and handling, virological EMBs analyses, and participated in manuscript editions. UR was involved in the design of the TF gene assay, data analysis, and participated in manuscript editions. RH and HL contributed by selection of explanted heart specimens and editing the manuscript. HDV supervised the overall design and coordination of the study, reviewed the data, and edited the manuscript. KaKo designed the gene assays for HPRT-CCM, and of the remaining self-designed non-TRBV/TRBC gene assays (except for TF), and was involved in the design and coordination the study, establishing the SSRT-PreAmp technique, was involved in the statistical analysis, and she wrote parts of and edited the manuscript. All authors read and approved the final manuscript.

## Supplementary Material

Additional file 1 T-PreAmp real-time RT-PCR gene expression results of all investigated genes in EMBs from DCM and DCMi patients related to HPRT-CCM. Gene expression (E; normalized to HPRT-CCM) T-PreAmp real-time RT-PCR results in EMBs from DCM patients compared with EMBs from patients with immunohistologically confirmed DCMi (>7 CD3^+ ^infiltrates/mm^2^). The values are given as means ± SD, followed by the respective p values in significantly different comparisons. Non-significant comparisons are denoted as n.s.. All investigated genes were quantified out of one single T-PreAmp reaction per EMB. Click here for file

Additional file 2 T-PreAmp real-time RT-PCR gene expression results of all investigated genes in EMBs from DCM and DCMi patients related to CDKN1B. Gene expression (E; normalized to CDKN1B) T-PreAmp real-time RT-PCR results in EMBs from DCM patients compared with EMBs from patients with immunohistologically confirmed DCMi (>7 CD3^+ ^infiltrates/mm^2^). The values are given as means ± SD, followed by the respective p values in significantly different comparisons. Non-significant comparisons are denoted as n.s.. All investigated genes were quantified out of one single T-PreAmp reaction per EMB. Click here for file

Additional file 3 List of abbreviations. The table enlists abbreviations commonly used in this manuscript. Click here for file
