# Supplementary material for: Preamplification techniques for real-time RT-PCR analyses of endomyocardial biopsies
Source: BMC Mol Biol. 2008 Jan 14;9:3. doi: 10.1186/1471-2199-9-3 (PMC2262094; doi:10.1186/1471-2199-9-3)
Supplement: Additional file 1 — T-PreAmp real-time RT-PCR gene expression results of all investigated genes in EMBs from DCM and DCMi patients related to HPRT-CCM. Gene expression (E; normalized to HPRT-CCM) T-PreAmp real-time RT-PCR results in EMBs from DCM patients compared with EMBs from patients with immunohistologically confirmed DCMi (>7 CD3+ infiltrates/mm2). The values are given as means ± SD, followed by the respective p values in significantly different comparisons. Non-significant comparisons are denoted as n.s.. All investigated genes were quantified out of one single T-PreAmp reaction per EMB. [file 1471-2199-9-3-S1.doc]

#### Additional file 1: T-PreAmp real-time RT-PCR gene expression results of all investigated genes in EMBs from DCM and DCMi patients related to HPRT-CCM

| **Gene** | **E / HPRT-CCM**  **DCM** | **E / HPRT-CCM**  **DCMi** | **p value** |
| --- | --- | --- | --- |
| **CDKN1B** | 3.12755+1.57223 | 3.73352+1.68606 | n.s. |
| **CD3d** | 0.014163+0.008761 | 0.045395+0.015252 | <0.0001 |
| **CD3z** | 0.043139+0.010119 | 0.060631+0.007718 | 0.0023 |
| **TRBC** | 0.015027+0.010150 | 0.027622+0.010532 | 0.0098 |
| **TRBV2** | 0.001487+0.001300 | 0.004503+0.002881 | 0.0053 |
| **TRBV3** | 0.002057+0.001900 | 0.003093+0.001438 | n.s. |
| **TRBV4** | 0.001501+0.001432 | 0.005441+0.003850 | 0.0047 |
| **TRBV5** | 0.002831+0.001945 | 0.003372+0.001318 | n.s. |
| **TRBV6** | 0.002926+0.002154 | 0.005478+0.002562 | 0.0307 |
| **TRBV7** | 0.000171+0.000123 | 0.000499+0.000628 | n.s. |
| **TRBV9** | 0.001394+0.000978 | 0.00298+0.003142 | n.s. |
| **TRBV10** | 0.000186+0.000127 | 0.000905+0.000967 | 0.0245 |
| **TRBV11** | 0.001264+0.000845 | 0.002763+0.002110 | n.s. |
| **TRBV12** | 0.006053+0.008008 | 0.008183+0.005067 | n.s. |
| **TRBV13** | 0.000763+0.000856 | 0.000724+0.000544 | n.s. |
| **TRBV14** | 0.000452+0.000543 | 0.000806+0.000679 | n.s. |
| **TRBV15** | 0.000163+0.000113 | 0.001143+0.001657 | n.s. |
| **TRBV16** | 0.000067+0.000070 | 0.000064+0.000035 | n.s. |
| **TRBV18** | 0.001177+0.000969 | 0.002859+0.002871 | n.s. |
| **TRBV19** | 0.005453+0.00884 | 0.006189+0.005485 | n.s. |
| **TRBV20** | 0.003935+0.003140 | 0.019818+0.018318 | 0.0105 |
| **TRBV23** | 0.000408+0.000269 | 0.006402+0.008324 | 0.0270 |
| **TRBV24** | 0.000282+0.000265 | 0.004754+0.006232 | 0.0075 |
| **TRBV25** | 0.000201+0.000147 | 0.000765+0.00085 | n.s. |
| **TRBV27** | 0.000522+0.000428 | 0.000870+0.000652 | n.s. |
| **TRBV28** | 0.003027+0.003362 | 0.004337+0.003937 | n.s. |
| **TRBV29** | 0.001302+0.001168 | 0.002815+0.00138 | 0.0192 |
| **TRBV30** | 0.001026+0.003524 | 0.003524+0.003524 | n.s. |
| **IL1b** | 0.013854+0.016054 | 0.012714+0.004540 | n.s. |
| **IL2** | 0.000102+0.000071 | 0.000144+0.000092 | n.s. |
| **IL5** | 0.000038+0,000029 | 0.000064+0,000047 | n.s. |
| **IL6** | 0.002020+0.001246 | 0.004133+0.002452 | 0.0094 |
| **IL10** | 0.001182+0.000779 | 0.004259+0.000316 | n.s. |
| **IL27** | 0.002125+0.001724 | 0.004983+0.009014 | n.s. |
| **IFNb** | 0.001815+0.001220 | 0.001300+0.000648 | n.s. |
| **IFNg** | 0.000193+0.000158 | 0.000343+0.000181 | n.s. |
| **TNFa** | 0.028511+0.014230 | 0.052024+0.017001 | 0.0049 |
| **TGFb** | 1.30737+0.30945 | 1.63956+1.19337 | n.s. |
| **CXCL14** | 0.151853+0.145437 | 0.054158+0.052000 | 0.0299 |
| **CX3CL1** | 0.96874+0.431717 | 1.39651+0.790158 | 0.0245 |
| **APN** | 0.390377+0.555952 | 0.035850+0.058782 | 0.0092 |
| **APN-R1** | 3.53288+0.45195 | 4.99193+0.73804 | n.s. |
| **APN-R2** | 1.19250+0.55202 | 1.33647+1.07774 | n.s. |
| **NFATC3** | 1.29493+0.479396 | 2.56855+0.776956 | 0.0052 |
| **CYR61** | 0.85785+0.47640 | 2.94840+2.33843 | <.0001 |
| **TF** | 3.59608+1.01046 | 2.14158+0.53169 | 0.0109 |
| **Ku70** | 6.59481+2.22542 | 6.46788+1.66238 | n.s. |
| **Ku80** | 5.67282+2.03474 | 6.66986+2.80126 | n.s. |
| **FoxP3** | 0.003726+0.001700 | 0.004675+0.001079 | n.s. |
| **T-bet** | 0.014431+0.005826 | 0.022267+0.008127 | 0.0355 |
| **GATA3** | 0.036669+0.014794 | 0.035425+0.012985 | n.s. |
| **GRAIL** | 0.000360+0.000344 | 0.000882+0.000673 | n.s. |
| **Granzyme A** | 0.014704+0.006914 | 0.035028+0.011726 | 0.0004 |
| **Granzyme B** | 0.062487+0.022406 | 0.105532+0.026082 | 0.0027 |
| **Granulysin** | 0.206285+0.186560 | 0.532339+0.327002 | 0.0183 |
| **Perforin** | 0.174231+0.065640 | 0.222637+0.025587 | n.s. |
| **MAN1A2** | 1.71415+0.708471 | 1.25648+0.296617 | n.s. |
| **Eomesodermin** | 0.014428+0.007246 | 0.036024+0.007900 | 0.0011 |
| **RHAMM** | 0.003246+0.002201 | 0.002402+0.000121 | n.s. |
| **Rho GTPAse 1** | 0.025522+0.012113 | 0.024687+0.007481 | n.s. |
| **Rho GTPAse 2** | 0.095418+0.085820 | 0.087212+0.036425 | n.s. |
| **TLR3** | 0.283233+0.192424 | 0.175524+0.093475 | n.s. |
| **TLR4** | 0.119932+0.031982 | 0.097289+0.032813 | n.s. |
| **TLR7** | 0.033287+0.012271 | 0.035395+0.022657 | n.s. |
| **TLR8** | 0.002743+0.003041 | 0.001718+0.000684 | n.s. |
| **TLR9** | 0.026647+0.013467 | 0.019119+0.004807 | n.s. |
| **MYD88** | 0.338701+0.138731 | 0.363109+0.309805 | n.s. |
| **TRAF3** | 0.705678+0.141575 | 0.592868+0.276950 | n.s. |
| **TRAF6** | 0.283120+0.102816 | 0.219573+0.053252 | n.s. |
| **IRF3** | 0.929970+0.303027 | 0.749030+0.318643 | n.s. |
| **IRF7** | 0.243138+0.139050 | 0.156422+0.044073 | n.s. |
| **MAPK8** | 1.79372+0.16447 | 1.56295+0.15802 | n.s. |
| **MAPK14** | 2.55389+1.12504 | 2.30904+0.40677 | n.s. |
| **STAT1** | 2.79895+1.36936 | 2.23812+0.81226 | n.s. |
| **SOCS3** | 0.059504+0.103335 | 0.025227+0.022936 | n.s. |
| **AP-1** | 4.07092+2.48069 | 3.01740+0.87571 | n.s. |
| **HSP70** | 3.14543+0.88026 | 2.76154+1.07441 | n.s. |
| **Laminin** | 0.061661+0.030909 | 0.064347+0.036184 | n.s. |
| **Collagen I** | 2.26194+1.59476 | 1.71738+0.99188 | n.s. |
| **Collagen III** | 1.87043+1.30297 | 2.53042+1.48650 | n.s. |
| **Collagen IV** | 2.49675+0.949609 | 2.20450+0.773285 | n.s. |
| **MMP2** | 5.27479+3.61284 | 3.94349+1.55918 | n.s. |
| **MMP8** | 0.008292+0.012456 | 0.000689+0.001448 | n.s. |
| **MMP9** | 0.086567+0.02957 | 0.018575+0.02831 | n.s. |
| **TIMP1** | 3.38820+2.04022 | 2.45604+1.00193 | n.s. |
| **TIMP4** | 0.846876+0.401468 | 0.656733+0.291593 | n.s. |
| **uPA** | 0.082603+0.026158 | 0.072779+0.020283 | n.s. |
| **av5b1** | 1.04571+0.283760 | 1.71316+0.844310 | 0.0033 |
| **GDF15** | 0.020984+0.013187 | 0.041715+0.028283 | 0.0127 |
| **CD62E** | 0.002714+0.001093 | 0.012877+0.008890 | 0.0119 |
| **CD56** | 0.003084+0.001365 | 0.002686+0.001000 | n.s. |
