# Supplementary material for: Preamplification techniques for real-time RT-PCR analyses of endomyocardial biopsies
Source: BMC Mol Biol. 2008 Jan 14;9:3. doi: 10.1186/1471-2199-9-3 (PMC2262094; doi:10.1186/1471-2199-9-3)
Supplement: Additional file 2 — T-PreAmp real-time RT-PCR gene expression results of all investigated genes in EMBs from DCM and DCMi patients related to CDKN1B. Gene expression (E; normalized to CDKN1B) T-PreAmp real-time RT-PCR results in EMBs from DCM patients compared with EMBs from patients with immunohistologically confirmed DCMi (>7 CD3+ infiltrates/mm2). The values are given as means ± SD, followed by the respective p values in significantly different comparisons. Non-significant comparisons are denoted as n.s.. All investigated genes were quantified out of one single T-PreAmp reaction per EMB. [file 1471-2199-9-3-S2.doc]

#### Additional file 2: T-PreAmp real-time RT-PCR gene expression results of all investigated genes in EMBs from DCM and DCMi patients related to CDKN1B

| **Gene** | **E / CDKN1B**  **DCM** | **E / CDKN1B**  **DCMi** | **p value** |
| --- | --- | --- | --- |
| **HPRT-CCM** | 0.299240+0.045955 | 0.310031+0.112730 | n.s. |
| **CD3d** | 0.005313+0.003732 | 0.013202+0.003592 | <0.0001 |
| **CD3z** | 0.015746+0.004887 | 0.026458+0.005847 | 0.0062 |
| **TRBC** | 0.004886+0.002290 | 0.007917+0.002221 | 0.0050 |
| **TRBV2** | 0.000540+0.000401 | 0.001312+0.000675 | 0.0045 |
| **TRBV3** | 0.000775+0.000589 | 0.001102+0.000664 | n.s. |
| **TRBV4** | 0.000471+0.000376 | 0.000471+0.000901 | 0.0039 |
| **TRBV5** | 0.001023+0.000769 | 0.001066+0.000527 | n.s. |
| **TRBV6** | 0.001088+0.000896 | 0.001932+0.000751 | 0.0450 |
| **TRBV7** | 0.000074+0.000071 | 0.000144+0.000119 | n.s. |
| **TRBV9** | 0.108572+0.077673 | 0.108497+0.086295 | n.s. |
| **TRBV10** | 0.000081+0.000080 | 0.000269+0.000258 | 0.0336 |
| **TRBV11** | 0.000439+0.000296 | 0.000751+0.000438 | n.s. |
| **TRBV12** | 0.002041+0.002092 | 0.002505+0.001481 | n.s. |
| **TRBV13** | 0.000284+0.000345 | 0.000230+0.000122 | n.s. |
| **TRBV14** | 0.000189+0.000283 | 0.000257+0.000240 | n.s. |
| **TRBV15** | 0.000067+0.000063 | 0.000330+0.000433 | n.s. |
| **TRBV16** | 0.000038+0.000042 | 0.000020+0.000008 | n.s. |
| **TRBV18** | 0.000471+0.000443 | 0.000681+0.000544 | n.s. |
| **TRBV19** | 0.001797+0.002304 | 0.001888+0.001510 | n.s. |
| **TRBV20** | 0.001409+0.000978 | 0.006499+0.007088 | 0.0290 |
| **TRBV23** | 0.000134+0.000077 | 0.001520+0.001829 | 0.0208 |
| **TRBV24** | 0.000103+0.000109 | 0.001022+0.000972 | 0.0055 |
| **TRBV25** | 0.000082+0.000071 | 0.000189+0.000179 | n.s. |
| **TRBV27** | 0.000169+0.000098 | 0.000264+0.000226 | n.s. |
| **TRBV28** | 0.000944+0.000925 | 0.001511+0.001434 | n.s. |
| **TRBV29** | 0.000460+0.000343 | 0.001011+0.000610 | 0.0248 |
| **TRBV30** | 0.000302+0.000307 | 0.000966+0.000952 | n.s. |
| **IL1b** | 0.004484+0.005270 | 0.003918+0.001778 | n.s. |
| **IL2** | 0.000040+0.000028 | 0.000044+0.000029 | n.s. |
| **IL5** | 0.000013+0,000009 | 0.000018+0.000012 | n.s. |
| **IL6** | 0.000652+0.000427 | 0.001199+0.000569 | 0.0276 |
| **IL10** | 0.000444+0.000277 | 0.000383+0.000114 | n.s. |
| **IL27** | 0.000697+0.000544 | 0.001469+0.002114 | n.s. |
| **IFNb** | 0.000539+0.000373 | 0.000408+0.000124 | n.s. |
| **IFNg** | 0.000077+0.000082 | 0.000167+0.000103 | n.s. |
| **TNFa** | 0.009038+0.004486 | 0.016878+0.010029 | 0.0165 |
| **TGFb** | 0.446927+0.100948 | 0.694076+0.345378 | n.s. |
| **CXCL14** | 0.044727+0.039035 | 0.016396+0.014341 | 0.0197 |
| **CX3CL1** | 0.310451+0.136292 | 0.480034+0.317710 | 0.0170 |
| **APN** | 0.109132+0.143038 | 0.011442+0.017685 | 0.0057 |
| **APN-R1** | 1.09161+0.241260 | 1.43347+0.853256 | n.s. |
| **APN-R2** | 0.363759+0.133625 | 0.427120+0.332245 | n.s. |
| **NFATC3** | 0.339242+0.117685 | 0.722471+0.255529 | 0.0063 |
| **CYR61** | 0.275270+0.138329 | 0.927302+0.688838 | <.0001 |
| **TF** | 0.927613+0.127482 | 0.609946+0.235874 | 0.0158 |
| **Ku70** | 1.98543+0.321460 | 2.15458+0.580389 | n.s. |
| **Ku80** | 1.69723+0.478514 | 2.03516+0.539191 | n.s. |
| **FoxP3** | 0.001458+0.000992 | 0.002031+0.000569 | n.s. |
| **T-bet** | 0.004867+0.001658 | 0.009905+0.004872 | 0.0275 |
| **GATA3** | 0.014280+0.004380 | 0.015729+0.008063 | n.s. |
| **GRAIL** | 0.002158+0.002814 | 0.002076+0.001870 | n.s. |
| **Granzyme A** | 0.006380+0.002616 | 0.016283+0.003742 | 0.0003 |
| **Granzyme B** | 0.024523+0.010114 | 0.047780+0.011263 | 0.0038 |
| **Granulysin** | 0.119557+0.050672 | 0.279193+0.127406 | 0.0124 |
| **Perforin** | 0.075309+0.00788 | 0.093023+0.00932 | n.s. |
| **MAN1A2** | 0.532779+0.203600 | 0.398876+0.143717 | n.s. |
| **Eomesodermin** | 0.004603+0.003034 | 0.010941+0.002335 | 0.0041 |
| **RHAMM** | 0.001071+0.001039 | 0.000742+0.000141 | n.s. |
| **Rho GTPAse 1** | 0.008398+0.004888 | 0.007812+0.003404 | n.s. |
| **Rho GTPAse 2** | 0.026812+0.019056 | 0.026619+0.011481 | n.s. |
| **TLR3** | 0.076626+0.050145 | 0.058470+0.035159 | n.s. |
| **TLR4** | 0.457398+0.196947 | 0.385117+0.094412 | n.s. |
| **TLR7** | 0.009547+0.004128 | 0.011503+0.006589 | n.s. |
| **TLR8** | 0.000767+0.000837 | 0.000581+0.000263 | n.s. |
| **TLR9** | 0.076597+0.057265 | 0.055819+0.016446 | n.s. |
| **MYD88** | 0.094490+0.020615 | 0.108869+0.057315 | n.s. |
| **TRAF3** | 0.217706+0.046471 | 0.188967+0.041840 | n.s. |
| **TRAF6** | 0.080386+0.015606 | 0.075971+0.015030 | n.s. |
| **IRF3** | 0.261461+0.042617 | 0.240426+0.048446 | n.s. |
| **IRF7** | 0.067211+0.00666 | 0.052814+0.00613 | n.s. |
| **MAPK8** | 0.592280+0.139968 | 0.521065+0.119595 | n.s. |
| **MAPK14** | 0.757344+0.125538 | 0.676438+0.128872 | n.s. |
| **STAT1** | 0.804401+0.231204 | 0.840088+0.500526 | n.s. |
| **SOCS3** | 0.019162+0.038797 | 0.008718+0.007042 | n.s. |
| **AP-1** | 1.08880+0.538690 | 1.02269+0.314771 | n.s. |
| **HSP70** | 1.00170+0.493614 | 0.95446+0.318767 | n.s. |
| **Laminin** | 0.017334+0.008134 | 0.020180+0.008664 | n.s. |
| **Collagen I** | 0.666100+0.511388 | 0.615157+0.424001 | n.s. |
| **Collagen III** | 0.620918+0.460926 | 0.760717+0.520429 | n.s. |
| **Collagen IV** | 0.735370+0.362891 | 0.752105+0.304101 | n.s. |
| **MMP2** | 1.40084+0.808405 | 1.36931+0.518494 | n.s. |
| **MMP8** | 0.002316+0.003422 | 0.000230+0.000435 | n.s. |
| **MMP9** | 0.029770+0.041028 | 0.006372+0.006333 | n.s. |
| **TIMP1** | 0.869000+0.387152 | 0.804672+0.253732 | n.s. |
| **TIMP4** | 0.231838+0.096896 | 0.214803+0.085051 | n.s. |
| **uPA** | 0.023840+0.007039 | 0.024350+0.008948 | n.s. |
| **av5b1** | 0.324506+0.088420 | 0.537818+0.296695 | 0.0061 |
| **GDF15** | 0.007592+0.005663 | 0.013882+0.009277 | 0.0348 |
| **CD62E** | 0.000879+0.000591 | 0.003719+0.002605 | 0.0187 |
| **CD56** | 0.000800+0.000334 | 0.000825+0.000260 | n.s. |
