# Supplementary material for: Preamplification techniques for real-time RT-PCR analyses of endomyocardial biopsies
Source: BMC Mol Biol. 2008 Jan 14;9:3. doi: 10.1186/1471-2199-9-3 (PMC2262094; doi:10.1186/1471-2199-9-3)
Supplement: Additional file 3 — List of abbreviations. The table enlists abbreviations commonly used in this manuscript. [file 1471-2199-9-3-S3.doc]

#### Additional file 2: List of abbreviations

| APN | adiponectin |
| --- | --- |
| CDKN1B | cyclin-dependent kinase inhibitor 1B |
| CYR61 | cysteine-rich angiogenic inducer 61 |
| CV | coefficient of variation |
| Ct | threshold cycle |
| E | expression of a target gene normalized to the housekeeping gene HPRT-CCM or CDKN1B applying the formula 2^-ΔCt^ |
| EMBs | endomyocardial biopsies |
| GDF15 | growth-differentiation factor 15 |
| HPRT | hypoxanthine-guanine phosphoribosyltransferase |
| HPRT-ABI | ABI inventoried Taqman® gene assay for HPRT |
| HPRT-CCM | HPRT gene assay designed by the Institute of Medical Immunology at the Charité-Campus Mitte |
| IL | interleukin |
| MGB | minor groove binder |
| MMP | matrix metalloproteinase |
| NFATC3 | nuclear factor of activated T-cells |
| PBMCs | peripheral blood mononuclear cells |
| PreAmp | preamplification |
| RT | reverse transcription |
| SSRT-PreAmp | multiplex preamplification procedure following a sequence specific reverse transcription |
| TE | Tris-EDTA |
| TIMP | tissue inhibitor of metalloproteinases |
| TF | tissue factor |
| TNFa | tumor necrosis factor alpha |
| T-PreAmp | preamplification using the Taqman® PreAmp Master Mix |
| TLR | Toll-like receptor |
| TRBC | T-cell receptor V constant (region) |
| TRBV | T-cell receptor V beta (region) |
| uPA | urokinase-type plasminogen activator |
